# Rational Design of Novel Inhibitors: Integrating 3D‐QSAR and Molecular Dynamics

**DOI:** 10.1155/bmri/4138899

**Published:** 2026-01-31

**Authors:** Neda Shakour, Aida Fayyaz, Farzin Hadizadeh, Saghi Sepehri

**Affiliations:** ^1^ Dental Research Center, Avicenna Institute of Clinical Sciences, Hamadan University of Medical Sciences, Hamadan, Iran, umsha.ac.ir; ^2^ Medicinal Plants and Natural Products Research Center, Hamadan University of Medical Sciences, Hamadan, Iran, umsha.ac.ir; ^3^ Department of Medicinal Chemistry, School of Pharmacy, Mashhad University of Medical Sciences, Mashhad, Iran, mums.ac.ir; ^4^ Department of Medicinal Chemistry, School of Pharmacy, Ardabil University of Medical Sciences, Ardabil, Iran, arums.ac.ir; ^5^ Students Research Committee, School of Pharmacy, Ardabil University of Medical Sciences, Ardabil, Iran, arums.ac.ir; ^6^ Biotechnology Research Center, Mashhad University of Medical Sciences, Mashhad, Iran, mums.ac.ir; ^7^ Pharmaceutical Sciences Research Center, Ardabil University of Medical Sciences, Ardabil, Iran, arums.ac.ir

**Keywords:** 3D-QSAR, ADMET, druglikeness, *Helicobacter pylori*, molecular docking, molecular docking simulation, pharmacophore modeling, triazole derivatives, urease inhibitors, virtual screening

## Abstract

This study presents an integrated computational framework that combines three‐dimensional quantitative structure–activity relationship (3D‐QSAR) modeling with molecular dynamics (MD) simulations to advance the rational design of triazole‐based urease inhibitors. Given the role of urease as a central virulence factor in *Helicobacter pylori* and its increasing relevance in antibiotic‐resistant infections, effective predictive methodologies are essential for early‐stage inhibitor development. A dataset of 54 triazole derivatives was examined using comparative molecular field analysis (CoMFA) and comparative molecular similarity indices analysis (CoMSIA), generating statistically robust and predictive models that elucidate the steric, hydrophobic, and hydrogen‐bonding features underlying inhibitory potency. These insights, supported by molecular docking and structure–activity relationship analyses, informed the construction of a pharmacophore model used to screen the ZINC database. Several candidate molecules, including ZINC84668437, ZINC84669798, and ZINC244633273, emerged as computationally promising and demonstrated favorable predicted druglikeness and ADMET characteristics. MD simulations were subsequently employed to evaluate the dynamic stability and conformational behavior of the ligand–urease complexes, reinforcing the coherence of the integrated computational workflow. The primary contribution of this work resides in its methodological integration of complementary in silico approaches rather than in experimental validation. Although the findings offer mechanistic insights and prioritize potential lead compounds, they remain predictive and necessitate future empirical confirmation. Overall, the study establishes a rigorous and academically grounded computational strategy that can guide subsequent efforts in the design of selective urease inhibitors for *H. pylori*–associated diseases.

## 1. Introduction

*Helicobacter pylori* is a major etiological agent of gastric diseases, including chronic gastritis, peptic ulcers, mucosa‐associated lymphoid tissue lymphoma, and gastric carcinoma [1]. With global infection rates ranging from 10% to 94% [2], *H. pylori* continues to pose a significant health burden. Although many infections remain asymptomatic, persistent colonization of the gastric mucosa can progressively damage epithelial tissues, leading to severe gastrointestinal disorders [3]. A key virulence factor in *H. pylori* is urease, a nickel‐dependent enzyme that hydrolyzes urea to ammonia and carbon dioxide, thereby neutralizing gastric acidity and enabling bacterial survival in the stomach [4–6]. Given its central role in pathogenesis, urease represents an attractive therapeutic target. Current treatments, typically combining antibiotics with bismuth complexes or proton pump inhibitors, are increasingly challenged by rising antibiotic resistance, high costs, and associated side effects [7, 8]. These limitations have intensified interest in identifying new classes of potent and selective urease inhibitors.

**Figure 1:**
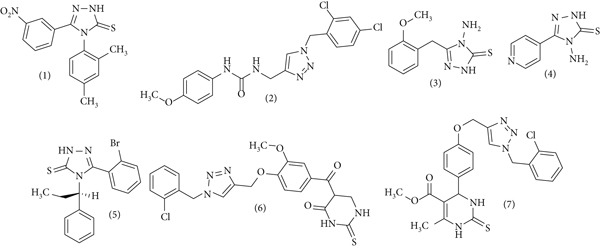
Schematic representation of selected chemical structures of urease inhibitors.

Several compound classes, such as hydroxamic acids and morpholines, have demonstrated promising urease inhibition, emphasizing the importance of metal‐chelating and hydrophobic interactions [9, 10] (Figure 1). Triazoles, in particular, have emerged as versatile scaffolds in drug design due to their favorable physicochemical characteristics and ability to participate in hydrogen bonding and dipole–dipole interactions [11]. Many triazole‐based molecules, including some in clinical use, have exhibited notable biological and urease‐inhibitory activities [12, 13].

Computational strategies now play a central role in accelerating drug discovery. Virtual screening (VS), molecular docking, molecular dynamics (MD) simulations, and quantitative structure–activity relationship (QSAR) modeling help identify and optimize potential inhibitors prior to synthesis [14–17]. Three‐dimensional QSAR approaches such as comparative molecular field analysis (CoMFA) and comparative molecular similarity indices analysis (CoMSIA) are particularly effective for elucidating steric, electrostatic, hydrophobic, and hydrogen‐bonding features governing biological activity [18–21]. Recent 3D‐QSAR studies on diverse urease inhibitor scaffolds have highlighted the value of these methods in guiding rational design [22].

**Figure 2:**
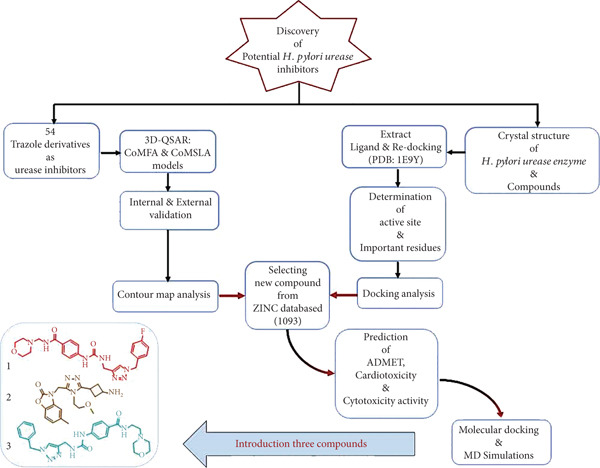
Illustrative summary of research outcomes.

In this study, we investigate triazole‐based urease inhibitors using an integrated VS framework. A dataset of 54 synthesized triazole analogues [1, 23] was analyzed using CoMFA and CoMSIA to identify key molecular determinants of activity. These findings were combined with molecular docking–derived structure–activity relationships (SARs) to generate a pharmacophore model. Pharmacophore‐based VS of the ZINC database was then performed to identify novel candidates, followed by assessment of druglikeness, ADMET profiles, and in silico cytotoxicity. Finally, MD simulations were used to evaluate the stability of ligand–urease complexes. This integrated approach is aimed at identifying promising urease inhibitors and support the development of targeted therapies against *H. pylori* infections (Figure 2).

## 2. Results and Discussion

### 2.1. 3D‐QSAR Model Study

3D‐QSAR has emerged as a pivotal predictive tool in pharmaceutical design, significantly enhancing the efficiency of drug development processes [24]. Although the traditional trial‐and‐error approach in drug discovery cannot be entirely eliminated, QSAR methodologies effectively reduce the number of synthesized molecules by identifying the most promising candidates early in the process [25]. This study rigorously examined various molecular descriptors to elucidate the factors influencing IC_50_ values, capitalizing on the advantages of a 3D‐QSAR model that incorporates the positional effects of functional groups on urease inhibitors.

A significant contribution to this domain was made by Ul‐Haq et al. who conducted a comprehensive 3D‐QSAR analysis on 30 bis‐coumarine derivatives. Their work successfully correlated chemical structures with urease inhibitory activity, demonstrating the efficacy of 3D‐QSAR in establishing meaningful relationships between molecular architecture and biological efficacy. The analysis yielded substantial *q* ^2^ and *r* ^2^ values through the CoMFA approach, highlighting the statistical relevance of this compound class [22].The use of CoMFA was particularly valuable in the absence of detailed mechanistic insights.

Further investigations into barbituric acid derivatives led to the development of robust CoMSIA and CoMFA models utilizing MMFF94 charges. These models achieved significant cross‐validation results, with predictive capabilities validated by ${r^{2}}_{\text{pred}}$ values exceeding 0.8 [26]. Additionally, a 3D‐QSAR analysis of 35 hydroxamic acid derivatives revealed that steric factors had a more pronounced impact on inhibitory activity compared with electrostatic factors. The generated CoMSIA models reinforced this observation, underscoring the critical roles of steric, electrostatic, and hydrophobic properties in urease inhibition [27]. In a separate investigation, Yang et al. focused on oxoindoline analogues as inhibitors of *H. pylori* urease, successfully identifying a potent compound with low cytotoxicity. Their study constructed a 3D‐QSAR model to explore SARs and provide valuable insights for future modifications [28]. Moreover, a 3D‐QSAR study on 30 bis‐coumarin analogues, recognized for their urease inhibitory properties, demonstrated a strong correlation between predicted and actual inhibitory activity, further validating the utility of this modeling approach [29]. Collectively, the CoMFA and CoMSIA contour map analyses presented in these studies offered critical insights into potential modifications of analogues for enhanced biological activity.

In the current investigation, a 3D‐QSAR analysis of triazole derivatives as urease inhibitors is presented, utilizing a dataset of 54 compounds. The developed models exhibit robust statistical parameters, including high *q* ^2^ and *r* ^2^ values, indicating excellent predictive power. The contour maps generated from CoMSIA and CoMFA analyses provide essential insights into the spatial requirements for biological activity, emphasizing the significance of electrostatic and steric interactions in the binding affinity of triazole derivatives to urease.

#### 2.1.1. Statistical Results Related to CoMFA

The statistical assessment of urease inhibitory activity for triazole derivatives is presented in Tables 1 and 2. The leave‐one‐out (LOO) cross‐validation yielded a q^2^ value of 0.664, whereas the cross‐validated coefficient ${{\text{r}}^{2}}_{\text{cv}}$ was 0.653, with the model incorporating three components (*N*) through partial least squares (PLS) analysis. These results indicate a strong predictive capability of the model. Further PLS analysis produced a noncross‐validated coefficient (${{\text{r}}^{2}}_{\text{ncv}}$) of 0.848, a standard error of estimate (SEE) of 0.228, and a Fisher ratio (*F*‐value) [30] of 74.108. These metrics confirm the model′s validity and robustness. Notably, the contributions to variance from steric and electrostatic fields were quantified at 1.141 and 1.526, respectively, suggesting that steric fields have a more significant impact on urease inhibitory activity. Moreover, the bootstrapped results from CoMFA indicated ${{\text{r}}^{2}}_{\text{bs}}$ and SEE_bs_ values of 0.848 and 0.219, respectively. These findings demonstrate minimal systematic errors and good internal consistency, further validating the model′s reliability in predicting the urease inhibitory activity of triazole derivatives.

**Table 1:** Statistical parameters of CoMFA and CoMSIA models using PLS analysis.

**Statistical Parameters**	**CoMFA**	**CoMSIA**
N	3	2
*q* ^2^ (LOO)	0.664	0.768
*r* ^2^	0.848	0.818
*r* ^2^ · *c* *v*	0.653	0.753
SEE	0.228	0.246
*F* value	74.108	91.893
${{\text{r}}^{2}}_{\text{pred}}$	0.800	0.808
${{\text{r}}^{2}}_{\text{bs}}$	0.848	0.833
SEE_bs_	0.219	0.232
SD_bs_	0.042	0.037
**Contributions**		
Steric	1.141	0.091
Electrostatic	1.526	0.120
Hydrophobic	1.526	0.120
Hydrogen bond donor	—	0.145
Hydrogen bond acceptor	—	0.173

*Note:* *N* is the optimal number of components, q^2^ is the leave‐one‐out (LOO) correlation coefficient, r^2^ is the noncross‐validation coefficient, ${{\text{r}}^{2}}_{\text{cv}}$ is cross‐validation coefficient, ${{\text{r}}^{2}}_{\text{pred}}$ is the predictive correlation coefficient of the test set, *F* is the *F*‐train value, ${{\text{r}}^{2}}_{\text{bs}}$ is the mean *r* ^2^ of boot strapping analysis, and SD_bs_ is the mean standard deviation by bootstrapping analysis.

Abbreviations: SEE, standard error of estimation; SEE_bs_, standard error of estimation of boot strapping analysis.

**Table 2:** Structures and predicted versus investigational inhibitory activities alongside residuals of the test set.

**Compound**	**p** **I** **C**_50_	**CoMFA**		**CoMSIA**	
		**Predicted**	**Residual**	**Predicted**	**Residual**
1a	3.7163	3.771	0.0546	3.704	−0.012
1f	4.87	4.871	0.0013	4.833	−0.0367
1j	4.61	4.922	0.3125	4.852	0.2422
1w	5.39	5.526	0.1363	5.33	−0.0601
2d	5.08	4.713	−0.3671	4.78	−0.2996
2e	5.09	5.469	0.3788	5.42	0.33
2h	4.99	4.91	−0.0795	4.82	−0.1696
2q	4.29	4.467	0.1772	4.555	0.2652
2p	4.35	4.916	0.5663	4.83	0.4797
2z	3.79	3.987	0.1968	4.049	0.2592

#### 2.1.2. Statistical Results Related to CoMSIA

The CoMSIA framework elucidates that variations in molecular properties are directly associated with alterations in ligand affinity [31]. This model is predicated on five fundamental fields: hydrogen bond donor (D), hydrogen bond acceptor (A), hydrophobic (H), steric (S), and electrostatic (E). A detailed summary of the statistical parameters pertinent to the CoMSIA model is provided in Table 1. For the model utilizing two components (*N* = 2), q^2^ ·  _LOO_ validation was determined to be 0.768, whereas ${{\text{r}}^{2}}_{\text{cv}}$ was recorded at 0.753. In the noncross‐validated PLS analysis, ${{\text{r}}^{2}}_{\text{ncv}}$ attained a value of 0.818, accompanied by a SEE of 0.246 and an *F*‐statistic of 91.893. The difference between *r* ^2^ and *q* ^2^ was calculated to be 0.05, indicating a robust model fit. Furthermore, a bootstrapped *r* ^2^ value of 0.833 reinforces the high degree of confidence in the analysis. The field descriptors generated from the CoMSIA model were quantified as follows: electrostatic (0.120), hydrophobic (0.991), steric (0.145), hydrogen bond donor (0.173), and hydrogen bond acceptor (0.309). It is noteworthy that these molecular fields demonstrate interdependencies, suggesting a complex interplay among the descriptors.

In the context of the 3D‐QSAR study, the IC_50_ values of the analogues were transformed into their respective pIC_50_ values (defined as ‐log IC_50_), which were employed as the dependent variable for the 3D‐QSAR model. The dataset was systematically divided into a test set comprising 10 compounds for the validation of the model′s predictive efficacy and a training set of 44 compounds for the construction of the QSAR model. The pIC_50_ values, predicted activity values (pIC_50_ pred), and the comparative activity differences between the CoMSIA and CoMFA models are delineated in Table S1.

#### 2.1.3. 3D‐QSAR Model Validation

The validation of the predictive power of the 3D‐QSAR models was conducted through three approaches:

##### 2.1.3.1. External Validation

**Figure 3:**
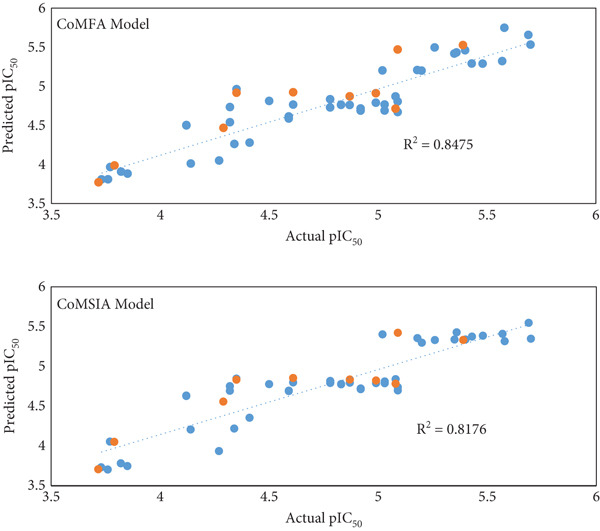
Plots of investigational against predicted pIC_50_ by CoMSIA and CoMFA (train set [blue], test set [orange]).

An external validation procedure was employed to rigorously assess the adequacy of the CoMSIA model. The validation yielded a score of 0.60, significantly exceeding the benchmark of 0.5. This result indicates that the model is robust and exhibits exceptional statistical predictive power. Additionally, all analogues were integrated into the model, with Figure 3 demonstrating the accuracy of the model′s predictions [30].

##### 2.1.3.2. Confirmation With Independent Test Set

Validation of the model′s predictive capabilities was conducted using an independent test set that was not involved in model development. The results indicated high stability and predictive power, as reflected by the q^2^ and r^2^ metrics, detailed in Table 1.

##### 2.1.3.3. *Y*‐Randomization Test

To evaluate the model′s robustness and minimize the risk of chance correlations, the *Y*‐randomization test was utilized for QSAR model validation (14, 15). This involved performing multiple random permutations of the biological activity (dependent variable) to create new QSAR models based on the original independent variable matrix. The resulting ranges for *r* ^2^ and *q* ^2^ values were 0.47 to 0.58 and −0.40 to −0.62 for CoMFA, and 0.41 to 0.56 and −0.42 to −0.63 for CoMSIA. These relatively low values suggest that the satisfactory results obtained in the final model construction were not due to chance [32, 33].

The differences in activity between the test and training compound sets for the CoMFA and CoMSIA models are illustrated in Figure S1. The CoMFA model identified 25 compounds with negative residual activity values and 29 compounds with positive deviations, whereas the CoMSIA model revealed 31 compounds with negative residual activity values and 23 compounds with positive deviations.

#### 2.1.4. Contour Maps

**Figure 4:**
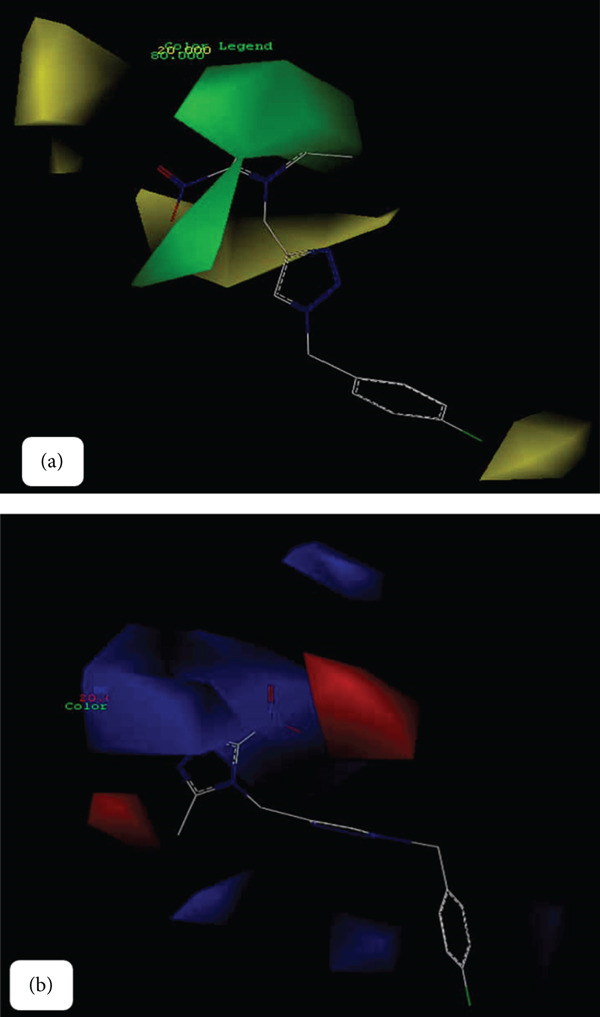
CoMFA contour maps of compound 1p (a) unfavorable (yellow) and favorable (green) steric field, (b) electronegative (red) and electropositive (blue) fields.

**Figure 5:**
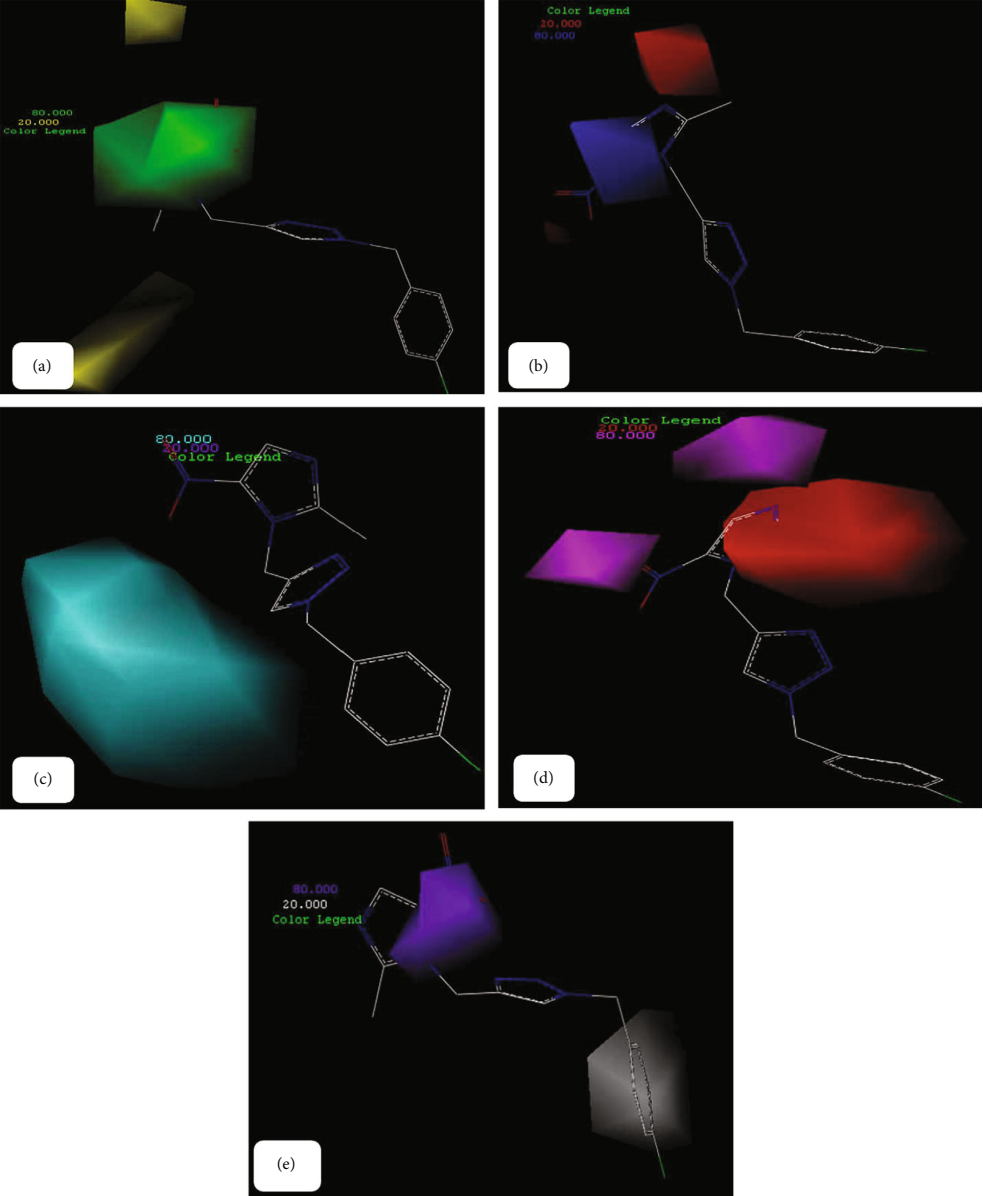
CoMsia contour maps of compound 1p (a) unfavorable (yellow) and favorable (green) steric field, (b) electronegative (red) and electropositive (blue) field, (c) unfavorable (purple) and favorable (cyan) hydrogen bond donor field, (d) unfavorable (red) and favorable (magenta) hydrogen bond acceptor field, (e) unfavorable (white) and favorable (purple) hydrophobic field.

A notable feature of the CoMFA and CoMSIA models is the generation of contour maps. These maps illustrate the varying influences of distinct groups on drug activity, which arise from different binding positions. The contour plots offer a detailed representation of these interactions, facilitating the design of pharmaceuticals with enhanced efficacy based on the insights derived from the maps [34]. In this study, we focused on compound 1p, which demonstrated the highest inhibitory activity against urease. The contours representing the hydrogen bond acceptor field, hydrogen bond donor field, electrostatic field, potential spatial field, and hydrophobic field from the CoMSIA model, alongside the contours of the potential electrostatic field and spatial field from the CoMFA model, were generated (Figures 4 and 5). The largest contours of the CoMSIA model are provided by the hydrogen bond acceptor field, followed by the hydrogen bond donor field.

Figure 4a illustrates the spatial arrangement of steric hindrance affecting the binding affinity of compound 1p. Areas highlighted in yellow indicate regions where steric clashes may reduce activity, whereas green areas suggest favorable steric interactions that enhance binding. Figure 4b displays the electrostatic potential around compound 1p. Red regions indicate areas of electronegativity that may attract positively charged groups, whereas blue areas represent electropositive regions that could interact favorably with negatively charged moieties.

Figure 5a illustrates the steric influences on compound 1p′s activity, emphasizing the regions that contribute positively or negatively to binding interactions. Figure 5b highlights the electrostatic properties surrounding compound 1p, identifying regions that can facilitate or hinder interaction with target sites. Figure 5c indicates the potential for hydrogen bond donation, with purple regions suggesting unfavorable interactions and cyan regions indicating favorable hydrogen bond donor characteristics. Figure 5d represents the hydrogen bond acceptor potential of compound 1p, where red areas denote unfavorable interactions, and magenta areas highlight favorable hydrogen bond acceptance. Figure 5e illustrates the hydrophobic characteristics of compound 1p, with white regions indicating areas that may disrupt hydrophobic interactions, whereas purple regions suggest favorable hydrophobic interactions.

### 2.2. Analyzing Docking Interactions in Specific Active Site

Bacterial ureases are heteromeric enzymes composed of three subunits: *α*, *β*, and *γ*. The *α*‐subunit contains a TIM (Triosephosphate isomerase) barrel and two Ni^2+^ centers, which are essential for its catalytic activity [9, 10]. All ureases require metal ions for activation, with Ni^2+^ being the most effective. A flexible flap region formed by cysteine residues stabilizes the catalytic transition state. Upon activation, this flap opens to facilitate the entry of urea, allowing for nucleophilic attack and the subsequent formation of carbamate and NH_3_. After catalysis, the flap reopens to release the product [35, 36]. The key residues involved in enzyme–compound interactions are Asp223, His248, Arg338, Asp362, Glu254, His274, His322, Glu222, Cys321, Met366, Ala169, Thr254, Gly279, His221, Gly280, and Ala365. The coordinated Ni^2+^ ions play a crucial role in facilitating the interaction between these key residues and the compounds [37–40].

**Figure 6:**
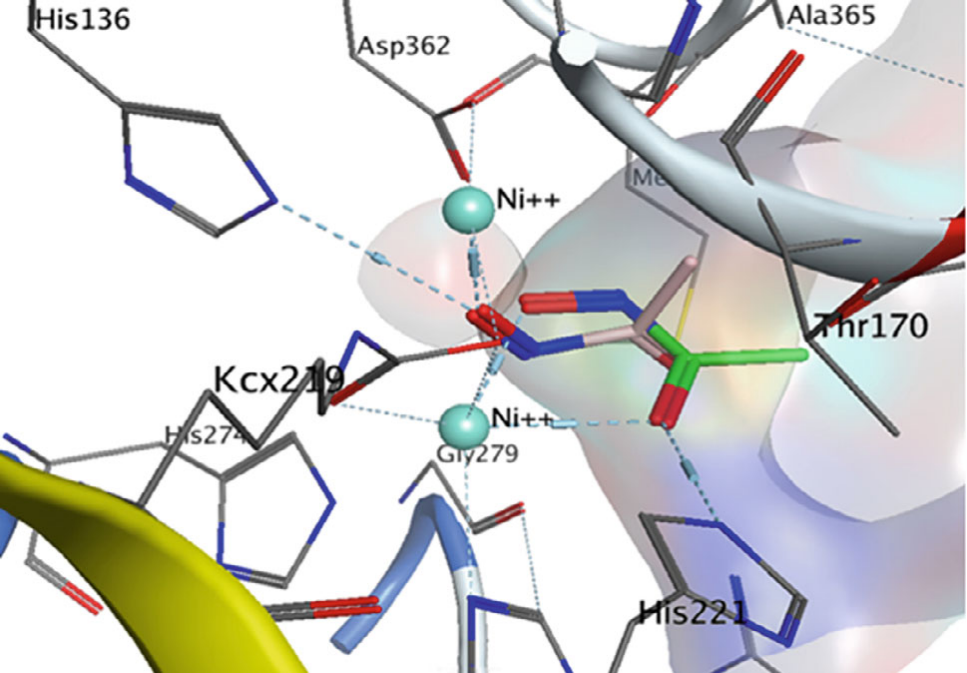
The overlay of acetohydroxamic acid finding from the x‐ray crystallography and acetohydroxamic acid finding from docking in the active site of urease enzyme.

To elucidate the binding modes of selected derivatives and validate QSAR findings, a docking study was conducted using Molecular Operating Environment (MOE) software on 54 compounds with known IC_50_ values against urease [23]. The cocrystal ligand acetohydroxamic acid was redocked into the *H. pylori* urease, yielding an RMSD of 1.47 Å, confirming the docking protocol′s reliability (Figure 6).

**Figure 7:**
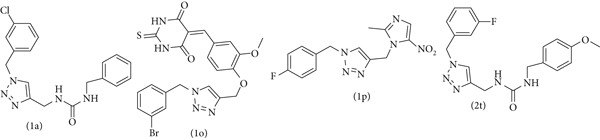
Structures of selected compounds.

From the docking results, four compounds were selected for further evaluation: 1p (most potent), 1o and 2t (moderately active), and 1a (least potent) (Figure 7). These compounds exhibited similar binding modes, interacting with key residues including Cys321, His322, Met366, Ala365, Arg338, Asn168, Asp223, and Ala169 (Figure S2). Notably, similar to the cocrystal ligand acetohydroxamic acid, the scaffolds of these derivatives (1a, 1o, 1p, and 2t) occupy the hydrophobic core of the urease active site. This indicates stabilization through hydrophobic interactions, as illustrated in the contour maps in Figures 4 and 5. Compounds 1p, 1o, and 2t formed interactions with residues His221, Gly279, and His248, whereas these residues did not interact with 1a. Importantly, compound 1p exhibited a significant interaction with Ni^2+^, akin to acetohydroxamic acid, whereas the other compounds did not interact with this metal ion (Figure S2).

Compounds 1p and 1o exhibited hydrogen bond interactions between Arg338 and Met366 and the NH group of the urea moiety and nitro group, respectively. Additionally, they formed a hydrogen bond–arene with Met366 and the triazole scaffold and with Cys321 and the phenyl ring. Compound 2t formed three hydrogen bonds with Arg368, Glu222, and Gly367 and with sulfur, Br, and NH of thiobarbiturate substitutes. It also exhibited a hydrogen bond–arene interaction with Met366 and the triazole scaffold. For compound 1a, the oxygen and NH groups of the urea moiety formed two hydrogen bonds with Cys321 and Arg338. The docking analysis reveals the binding mode of selected derivatives in the urease active site, highlighting the critical roles of His322, Met366, Cys321, and Arg338, along with significant hydrophobic interactions.

### 2.3. Design of New Analogues According to Ligand‐Based and Structure‐Based Studies

**Figure 8:**
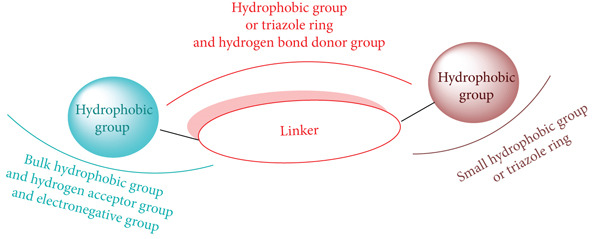
SAR revealed by molecular docking and 3D‐QSAR findings.

Building upon the insights gained from molecular docking analyses and the 3D‐QSAR model outcomes, molecule 1p was identified as a pivotal template for the design of novel triazole analogues, which were selected from the ZINC database. The SAR analysis presented in Figure 8 revealed that compounds featuring small hydrophobic groups or triazole rings, interconnected by an appropriate linker, exhibit enhanced inhibitory activity. Additionally, the presence of a linker moiety between two hydrophobic or (het) aromatic rings, particularly when one of these rings is hydrophobic or triazolic and includes a hydrogen donor group, significantly enhances the compounds′ biological efficacy. Importantly, the incorporation of a bulky hydrophobic group, paired with a hydrogen bond acceptor and an electronegative group on the opposite side of the linker, further amplifies the inhibitory potential against urea enzymes (Figure 8).

Leveraging this structural paradigm, a diverse set of approximately 1093 molecules, as presented in Table S2, was extracted from the ZINC database (https://zinc.docking.org/substances/home/). Subsequently, a blind docking‐based VS was performed using AutoDock Vina within the PyRx framework. The candidate compounds were meticulously ranked according to their predicted docking scores within the urease active site. This systematic ranking process led to the identification of 317 molecules that demonstrated more negative docking scores than compound 1p, positioning them for further evaluation in subsequent stages of the study.

### 2.4. Druglikeness and Absorption, Distribution, Metabolism, and Excretion (ADME) Analysis

The druglikeness profiles of the screened analogues were systematically evaluated according to Lipinski′s rule of five, utilizing the Molinspiration online platform. Lipinski′s criteria stipulate that compounds with optimal druglike characteristics should have no more than five hydrogen bond donors, a maximum of 10 hydrogen bond acceptors, a molecular weight below 500 Da, and a Log*P* value not exceeding 5. Departures from these parameters can adversely affect a compound′s oral bioavailability. To assess the hydrogen bonding potential of each compound, the number of nitrogen and oxygen atoms in their molecular structures was quantified. Additional physicochemical descriptors, such as the number of rotatable bonds (nRot) and the topological polar surface area (TPSA), were computed, with favorable thresholds defined as TPSA < 140 Å^2^ and nRot ≤ 10.

Among the 317 compounds evaluated, 285 were found to comply with Lipinski′s criteria, suggesting a high probability of satisfactory absorption and permeability across biological membranes.

Following this preliminary druglikeness screening, these 285 compounds were subjected to in silico pharmacokinetic (ADME) profiling using the SwissADME and admetSAR web‐based platforms. These computational tools enabled the prediction of the ADME characteristics, using the most active compound (compound 1p) as a reference. The ADME profiling included predictions of blood–brain barrier (BBB) permeability, human intestinal absorption (HIA), aqueous solubility, biodegradability, and carcinogenic potential. Additionally, interactions with key metabolic enzymes and transporters were evaluated, particularly concerning the potential for cytochrome P450 (CYP450) inhibition and the identification of P‐glycoprotein (P‐gp) substrates. From this thorough evaluation, six compounds were identified as optimal candidates, distinguished by their favorable ADME and druglikeness properties. The results for these six compounds are summarized in Tables 3 and S3.

**Table 3:** Calculations of druglikeness properties of selected compounds using Molinspiration server.

**ZINC_ ID**	**LogP**	**MW**	**HBD**	**HBA**	**nRotb**	**TPSA**
ZINC84668437	1.81	467.50	3	10	8	113.41
ZINC84669798	1.66	463.54	3	10	9	113.41
ZINC244633273	0.22	357.41	2	8	6	101.12
ZINC147991008	0.06	324.34	3	7	3	95.18
ZINC147991228	0.06	324.34	3	7	3	85.94
ZINC000148002556	0.09	294.31	3	6	2	85.94

Abbreviations: HBA, hydrogen bond acceptor; HBD, hydrogen bond donor; MW, molecular weight; nRotb, n‐rotatable bond; TPSA, surface belonging to polar atoms.

It should be noted that the ADMET analyses were conducted exclusively using computational predictions from web‐based tools. Accordingly, these results are preliminary and hypothetical, offering only a tentative assessment of pharmacokinetic potential. The inherent limitations of this computational method necessitate further experimental validation to confirm these findings.

### 2.5. In Silico Cardiotoxicity Prediction

Cardiotoxicity predictions were performed using the Pred‐hERG online service (http://www.labmol.com.br), which demonstrates a specificity, sensitivity, and accuracy of approximately 89%–90%. These predictions are vital for establishing a robust safety profile before any in vivo administration. The analysis revealed that the six compounds selected in the preceding step were found to be neither cardiotoxic nor hERG channel blockers, as presented in Table S4. This outcome underscores a favorable safety profile for these derivatives, thereby supporting their potential for further development in the drug discovery process.

### 2.6. In Silico Cytotoxicity Activity Prediction

The cytotoxic effects of the six selected compounds were evaluated using the cell line cytotoxicity prediction (CLC‐Pred) model. The analysis revealed that these compounds exhibited notable cytotoxicity against various cancer cell lines. Specifically, compounds ZINC000148002556, ZINC147991228, and ZINC147991008 demonstrated the highest levels of cytotoxic activity against the DMS‐114 lung carcinoma cell line, with predictive activity (*P* *a*) values exceeding 0.7. Further evaluation showed that compounds ZINC244633273, ZINC84669798, and ZINC84668437 were particularly effective against the NCI‐H838 non‐small cell lung carcinoma (*P* *a* = 0.446), Malme‐3M melanoma cell line (*P* *a* = 0.393), and ACHN papillary renal carcinoma cell line (*P* *a* = 0.354), respectively (Table 4.). Additionally, as detailed in Table 5, four compounds (ZINC000148002556, ZINC147991228, ZINC147991008, and ZINC244633273) demonstrated the highest activity on the HEL299 normal cell line, with *P* *a* values ranging from 0.166 to 0.183. Furthermore, compounds ZINC84669798 and ZINC84668437 exhibited significant activity on the peripheral blood mononuclear cell (PBMC) line, with *P* *a* values of 0.102 and 0.14, respectively. These results collectively indicate that none of the six compounds exhibited significant toxicity toward either the HEL299 or PBMC normal cell lines.

**Table 4:** Predictions of cytotoxicity activity compounds against cancer cell lines using CLC‐Pred.

**P** **a**	**P** **i**	**Cell line**	**Cell line full name**	**Tissue**	**Tumor type**
ZINC000148002556					
0.742	0.004	DMS‐114	Lung carcinoma	Lung	Carcinoma
0.666	0.009	HOP‐92	Non‐small cell lung carcinoma	Lung	Carcinoma
0.624	0.009	A498	Renal carcinoma	Kidney	Carcinoma
0.619	0.011	786‐0	Renal carcinoma	Kidney	Carcinoma
0.602	0.004	HOS	Osteosarcoma	Bone	Sarcoma
ZINC147991228					
0.718	0.004	DMS‐114	Lung carcinoma	Lung	Carcinoma
0.590	0.011	HOP‐92	Non‐small cell lung carcinoma	Lung	Carcinoma
0.591	0.012	A498	Renal carcinoma	Kidney	Carcinoma
ZINC147991008					
0.718	0.004	DMS‐114	Lung carcinoma	Lung	Carcinoma
0.590	0.011	HOP‐92	Non‐small cell lung carcinoma	Kidney	Carcinoma
0.591	0.012	A498	Renal carcinoma	Kidney	Carcinoma
ZINC244633273					
0.446	0.088	NCI‐H838	Non‐small cell lung carcinoma	Lung	Carcinoma
0.284	0.094	MDA‐MB‐231	Breast adenocarcinoma	Breast	Adenocarcinoma
0.279	0.106	HOS	Osteosarcoma	Bone	Sarcoma
ZINC84669798					
0.393	0.038	Malme‐3M	Melanoma	Skin	Melanoma
0.373	0.036	ACHN	Papillary renal carcinoma	Kidney	Carcinoma
0.325	0.009	MDA‐MB‐361	Breast adenocarcinoma	Breast	Adenocarcinoma
0.368	0.070	HT‐29	Colon adenocarcinoma	Colon	Adenocarcinoma
ZINC84668437					
0.354	0.042	ACHN	Papillary renal carcinoma	Kidney	Carcinoma
0.307	0.014	MDA‐MB‐361	Breast adenocarcinoma	Breast	Adenocarcinoma
0.346	0.053	Malme‐3M	Melanoma	Skin	Melanoma

**Table 5:** Predictions of cytotoxicity activity compounds on normal cell lines using CLC‐Pred.

**P** **a**	**P** **i**	**Cell line**	**Cell line full name**	**Tissue**
ZINC000148002556				
0.182	0.084	HEL299	Fibroblast	Lung
ZINC147991228				
0.183	0.083	HEL299	Fibroblast	Lung
ZINC147991008				
0.183	0.083	HEL299	Fibroblast	Lung
ZINC244633273				
0.166	0.100	HEL299	Fibroblast	Lung
ZINC84669798				
0.102	0.036	PBMC	Peripheral blood mononuclear cell	Blood
ZINC84668437				
0.141	0.022	PBMC	Peripheral blood mononuclear cell	Blood

It should be emphasized that the cytotoxicity predictions, derived exclusively from web‐based computational models, are preliminary and hypothetical. They provide only a tentative indication of potential biological activity, underscoring the need for experimental validation to confirm these findings and account for the limitations of in silico approaches.

### 2.7. Interactions of Optimal ZINC Compounds as Urease Inhibitors

The binding mechanisms of the newly identified compounds within the active site of urease were investigated using molecular docking simulations. The analysis indicated that these compounds interacted similarly to compound 1p, achieving superior docking scores (Table S5). Key amino acids, Cys321, Arg338, His322, Ala365, Asp362, His221, Met366, His248, Asp165, and Ala169, were identified as critical for binding, reducing the flexibility of the flap region and thereby decreasing enzymatic activity [36, 40–42].

The orientation and interactions of ZINC84668437 in the urease binding site (Figure S3) revealed that it formed hydrophobic interactions with residues such as His323 and Met317, while engaging in hydrogen bonding with Arg338, Cys321, and Asn168. This compound also exhibited H–arene interactions with Cys321 and chelated two Ni^2+^ ions through the oxygen atom of its morpholine ring.

ZINC84669798, shown in Figure S4, established four hydrogen bonds with Asn168, Cys321, Ala169, and His322, and engaged in hydrophobic interactions with multiple residues, including His323 and Asp165. It demonstrated nonbonded interactions with two Ni^2+^ ions but did not form *π*‐cation or H–arene interactions.

ZINC000244633273, depicted in Figure S5, was positioned in the binding pocket, forming hydrogen bonds with Arg338, Asp223, and Ala365, alongside H–arene interactions with Cys321. It also exhibited hydrophobic interactions with several residues and nonbonded interactions with Ni^2+^ ions.

The stereoisomers ZINC147991008 and ZINC147991228 displayed distinct interaction patterns; ZINC147991008 formed hydrogen bonds with Gln364, whereas ZINC147991228 interacted with Gly279. Both compounds showed hydrophobic contacts with various residues; notably, ZINC147991008 also displayed H–arene interactions with Arg338 (Figure S6).

The modification of ZINC147991228 by removing the methoxy group resulted in ZINC000148002556, which had the lowest docking score among the candidates. This compound formed three hydrogen bonds with Ala365, Arg338, and Gly279, compared with ZINC147991228′s single bond with Gly279. Both compounds displayed hydrophobic interactions with key residues, although ZINC000148002556 did not engage with some residues that ZINC147991228 did (Figure S7).

The findings indicate that the selected compounds effectively occupy the catalytic cavity, coordinating with Ni^2+^ ions and interacting with flap region residues, thereby inhibiting urease activity by limiting the flexibility of the mobile flap.

### 2.8. MD Simulations Study

MD simulations were employed to elucidate the activity of the *H. pylori* urease enzyme in response to binding with various compounds, as well as to evaluate the stability and interactions within the resulting compound–enzyme complexes. The analysis concentrated on three compounds with the highest docking scores (ZINC84668437, ZINC84669798, and ZINC244633273) (Table S4), alongside compound 1p (identified as the most potent compound), acetohydroxamic acid (an FDA‐approved drug and cocrystal ligand), and the apoprotein (devoid of a ligand). Each complex underwent a 100‐ns simulation, with the outcomes assessed through metrics including root mean square deviation (RMSD), potential energy, root mean square fluctuation (RMSF), radius of gyration (Rg), and hydrogen bond interactions.

Ligand flexibility is a critical factor in drug design, as flexible ligands can adopt multiple conformations, thereby enhancing their fit and facilitating optimal interactions, including hydrogen bonding. Although rigid ligands may achieve higher affinity when precisely aligned with the binding pocket, excessive rigidity can compromise adaptability and reduce binding efficacy [43–47]. The findings underscore the importance of achieving an optimal balance between flexibility and rigidity to enhance binding interactions and inform effective strategies in drug design.

#### 2.8.1. RMSD Analysis

**Figure 9:**
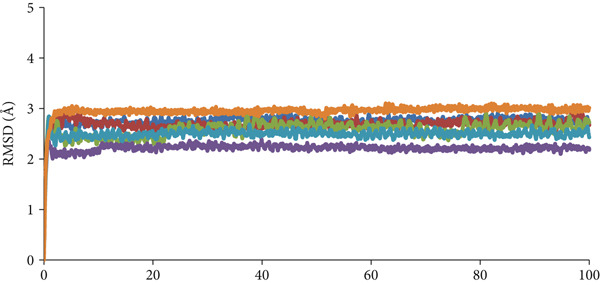
RMSD between *H. pylori* urease with compounds ZINC84668437 (purple), ZINC84669798 (light blue), ZINC244633273 (orange), 1p (green) as well as acetohydroxamic acid (Red), and apoprotein (dark blue) during MD simulations.

RMSD of the backbone atoms (C*α*, C, and *N*) is a critical metric for evaluating structural stability and was utilized to analyze overall simulation convergence and the equilibration of the compound–enzyme complexes. Figure 9 presents the RMSD plots for these complexes. The three candidate compounds demonstrated stability within the protein backbone, with the RMSD reaching a steady state at approximately 6.0 ns. Specifically, the RMSD values for the ZINC84668437–urease and ZINC84669798–urease complexes were both below 2.5 Å, whereas the ZINC244633273–urease complex exhibited an RMSD of less than 3 Å. Average RMSD values for the ZINC84668437, ZINC84669798, ZINC244633273, 1p, acetohydroxamic acid–urease, and apoprotein complexes were recorded at 2.21, 2.49, 2.95, 2.58, 2.68, and 2.75 Å, respectively. These results indicate that the ZINC84668437 and ZINC84669798 complexes with urease may exhibit stronger interactions with urease, contributing to their enhanced stability relative to the other complexes. Consequently, the ZINC84668437–urease complex displayed the lowest deviations among all tested structures. Throughout the MD simulations, all compounds exhibited minimal fluctuations, suggesting increased stability of the complexes. A fluctuation range of 1–3 Å is considered acceptable for small globular proteins [48, 49]. Overall, the candidate complexes demonstrated robust stability based on the average RMSD values, indicating that the structural integrity of the enzyme was maintained throughout the simulations.

#### 2.8.2. Potential Energy Measurement

A reliable approach to evaluate the stability of a compound–enzyme complex is through the measurement of potential energy. The potential energies of the compound–enzyme interactions for all complexes were assessed after a 100‐ns simulation, revealing fluctuations around −8311.09, −8211.04, −8174.14, −8211.04, and −8162.44 kcal/mol for the ZINC84668437, ZINC84669798, ZINC244633273, 1p, and acetohydroxamic acid complexes with urease, respectively (Figure S8). These findings demonstrate the stability of the complexes. Notably, the newly identified molecule ZINC84668437 exhibited the lowest potential energy, indicating enhanced activity and a strong binding affinity for urease. Compound ZINC84669798 displayed potential energy comparable with that of 1p, yet was lower than that of acetohydroxamic acid. In contrast, compound ZINC244633273 exhibited higher potential energy than all other compounds, except for acetohydroxamic acid.

#### 2.8.3. RMSF Analysis

RMSF is a vital metric for assessing macromolecular flexibility and reflects localized changes in protein structure [50]. This analysis involved calculating RMSF values, which quantify the divergence in molecular orientation from its initial conformation and the amplitude of motion among enzyme residues. The RMSF for each complex was computed using C*α* atoms. For all complexes, the fluctuation intensity remained below 3.0 Å, except for residues associated with loops or turns in the enzyme [51]. RMSF values were significantly higher in regions characterized by turns, loops, and coils when compared with sheet and helical structures [52]. This increase in RMSF values suggests greater flexibility and instability in binding within these regions [53].

**Figure 10:**
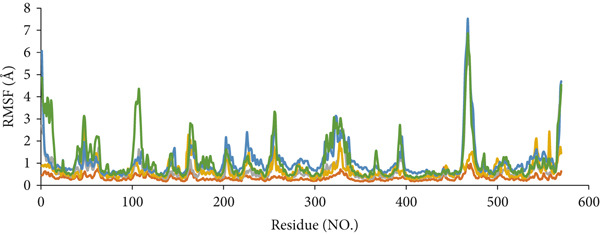
RMSF of the protein backbone atoms for each residue of ZINC244633273 (green), ZINC84669798 (blue), ZINC84668437 (yellow), 1p (gray) as well as acetohydroxamic acid (brown) urease complexes during MD simulations.

Figure 10 presents the RMSF plots for the ligand‐bound protein across all compounds. The enzyme structures of all complexes exhibited comparable RMSF distributions and dynamic characteristics. The average RMSF values for the complexes of ZINC244633273, ZINC84669798, ZINC84668437, 1p, and acetohydroxamic acid with urease were 0.74, 1.09, 1.11, 0.71, and 0.36 Å, respectively. The lower RMSF values observed for the compound–enzyme interactions involving residues such as Cys321, His221, Met366, Arg338, Ala365, and Asn168 suggest reduced flexibility due to stronger binding interactions with these amino acids. This study found that similar amino acids in these regions fluctuated across all derivatives, indicating their importance in binding. Residues exhibiting higher fluctuations were often situated away from the active site or within enzyme loops or terminals. Throughout the MD simulations, the complexes demonstrated stability, with nearly identical fluctuation patterns observed across all complexes. The average RMSF for all complexes was below 2.5 Å, with certain regions displaying high fluctuations whereas others showed minimal changes. Residues in the active site exhibited RMSF values of less than 4 Å, suggesting that this area is dense, rigid, and stable within a dynamic environment.

These findings indicate that the complexes ZINC84668437 and ZINC84669798 bind effectively and undergo minimal conformational changes compared with ZINC244633273. Acetohydroxamic acid and 1p exhibited smaller fluctuations than the candidate compounds, whereas ZINC84668437 displayed fluctuations comparable with those of acetohydroxamic acid and 1p. The RMSF results are consistent with previous studies, further supporting the stability and binding characteristics of these complexes [23, 40, 42].

#### 2.8.4. Assessment of Structural Compactness by Rg

**Figure 11:**
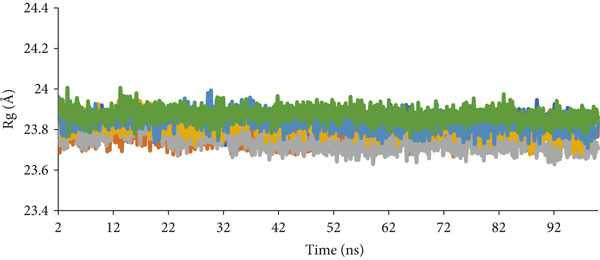
Rg plot of ZINC244633273 (green), ZINC84669798 (light blue), ZINC84668437 (yellow), 1p (gray), and acetohydroxamic acid (orange) urease complexes as well as apoprotein (dark blue) 0–100 ns.

Rg was employed to evaluate the compactness of the system. Elevated Rg values indicate a decrease in compactness and a more unfolded state [54], whereas lower values reflect increased compactness and structural rigidity, characteristic of a more folded conformation [55]. Average Rg values for the complexes involving ZINC244633273, ZINC84669798, ZINC84668437, 1p, and acetohydroxamic acid, along with the apoprotein, were measured at 23.87, 23.83, 23.82, 23.75, 23.77, and 23.83 Å, respectively. Throughout the MD simulations, these values exhibited minimal fluctuations, indicating stability in both the ligand–enzyme complexes and the apoprotein. This stability suggests that the backbone conformations of the enzyme remained unchanged following inhibitor binding, with molecular interactions not significantly altering the overall structure of urease (Figure 11).

The analysis revealed that the compounds did not induce substantial changes in the enzyme′s compactness. The modest fluctuations observed further corroborated the stability of the complexes, implying effective binding to the active site. All complexes exhibited robust values indicative of a highly folded enzyme–compound structure, suggesting that they enhance the rigidity and stability of the enzyme. The active site remains rigid, preventing significant conformational changes upon ligand binding.

#### 2.8.5. Hydrogen Bond Analysis of Complexes

The stability of the ligand–protein complex is maintained through various interactions, particularly hydrogen bonds, which are essential for structural integrity and ligand binding stability [56]. MD simulations indicated average hydrogen bond counts of 1.68, 1.64, 1.66, 0.07, and 0.86 for ZINC244633273, ZINC84669798, ZINC84668437, 1p, and acetohydroxamic acid with urease, respectively. Both hydrophobic and hydrogen bonds contributed to stabilizing the helical structure. During the simulations, ZINC244633273 established hydrogen bonds with Asp223 and His322, with lifespans of 60% and 100%. ZINC84668437 interacted with Asn168 and Asp165, whereas ZINC84669798 formed strong interactions with Cys321 and His138. ZINC244633273 also engaged with Glu222 and Thr170. Overall, the three compounds showed significant interactions with Asn168, Cys321, and Ala169, whereas Asp223, Met366, and Ala365 demonstrated key interactions with two compounds over the 100‐ns simulation (Figure S9).

## 3. Conclusion

This study represents a significant advancement in drug design through the integration of predictive QSAR methodologies, specifically 3D‐QSAR models, with MD simulations and molecular docking analyses. This approach has elucidated critical interactions between triazole derivatives and the urease enzyme, leading to the identification of several promising candidate inhibitors, including ZINC84668437, ZINC84669798, and ZINC244633273, which exhibit favorable druglike properties. It is important to clarify that the primary contribution of this research lies in the methodological integration of computational techniques rather than in experimental validation. Although the developed models demonstrate robust statistical validation, the therapeutic efficacy of the identified compounds requires further empirical testing to confirm their potential. This study establishes a foundational framework for future research focused on the synthesis and biological evaluation of the proposed analogues, highlighting the necessity of integrating computational insights with empirical methodologies. Such integration enhances the rational design of effective urease inhibitors and contributes to broader efforts in addressing urease‐related diseases.

## 4. Methods

### 4.1. 3D‐QSAR Model Study

#### 4.1.1. Optimization and Grouping of Triazole Derivatives

A total of 54 triazole derivatives were constructed using ChemOffice Bio 3D Ultra software (Version 12.0, Cambridge Soft Corporation, Cambridge, United Kingdom). The subsequent analyses were performed with Sybyl X‐1.2 software (Tripos, Inc., St. Louis, MO, United States, 2012) on a personal computer featuring an Intel Core i7 CPU. Computational models, specifically CoMSIA and CoMFA, were utilized to evaluate the derivatives. The initial geometries of these compounds were optimized through the MM2 molecular mechanics method, which is employed to accurately model the three‐dimensional structures and properties of molecular derivatives.

To calculate partial atomic charges, the Gasteiger–Huckel method was applied, whereas the Tripos force field was used for energy minimization of the compounds [57]. A logarithmic transformation of the IC_50_ values, measured in *μ*M, was conducted to derive pIC_50_ (‐log IC_50_). For external validation, the derivatives were randomly divided into two sets: a test set comprising 20% of the compounds (10 derivatives) and a training set comprising 80% (44 derivatives). Both sets exhibited structural diversity and encompassed a range of biological activities, ensuring a consistent distribution across high, medium, and low activity levels [33]. The study was approved by the Ardabil University Ethics Committee (IR.ARUMS.REC.1399.559).

#### 4.1.2. CoMFA and CoMSIA Analysis

After optimizing and classifying the triazole derivatives, the distill rigid force field was utilized to calculate aligned structures (Figure 12). Subsequently, CoMFA and CoMSIA analyses were performed. CoMSIA, a prominent 3D‐QSAR methodology, leverages known biological activity data to predict the efficacy of new pharmaceuticals [34]. It evaluates five features: steric, electrostatic, hydrogen bond acceptor, hydrophobic, and hydrogen bond donor properties, using a probe with a charge of +1.0, a radius of 1 Å, and parameters of +1.0 for hydrophobicity and hydrogen bonding [58].

Figure 12(a) Molecule 1p applied as a template to database alignment. (b) The aligned molecules in the training.

**Figure:**
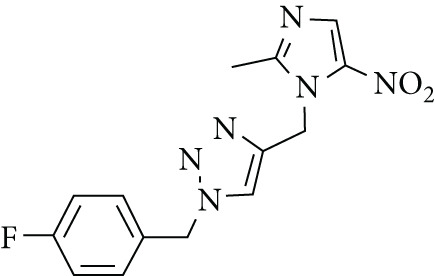
(a)

**Figure:**
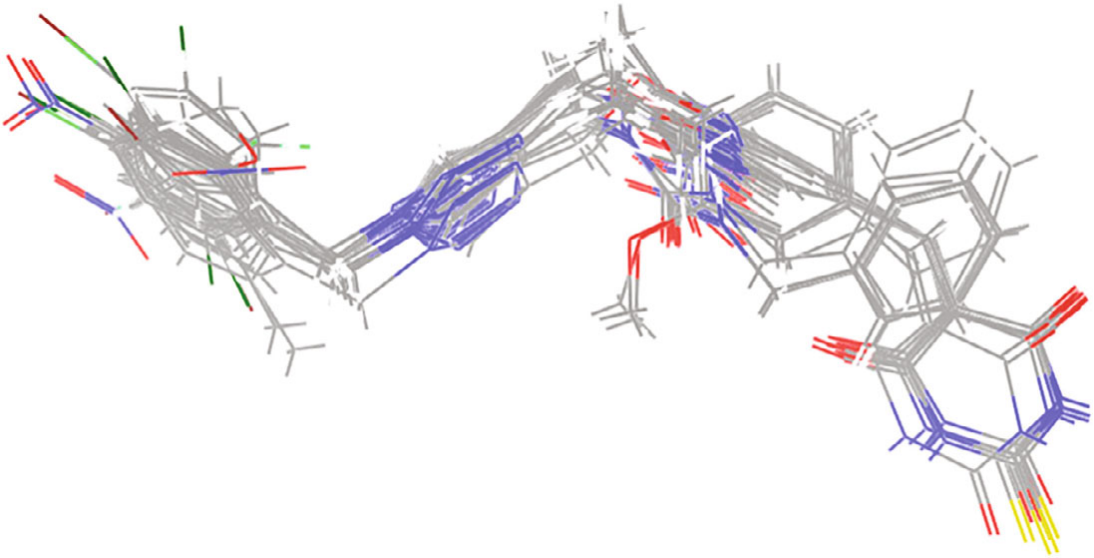
(b)

CoMFA aligns molecules sharing a common structural parent ring in three‐dimensional space, allowing for the calculation of interaction energies across various spatial coordinates [59]. The van der Waals radius of the sp^3^ carbon probe atom was set at 1.52 Å, with a charge of +1.0. Electrostatic and steric interactions were estimated using Coulomb and Lennard–Jones potentials, respectively, with an energy cutoff of 30 kcal/mol. A column filtering parameter of 2.0 kcal/mol was also applied to enhance analytical efficiency. Consequently, the PLS analysis demonstrated improved predictability and resolution, as indicated by cross‐validated (*q* ^2^) and correlation coefficient (*r* ^2^) metrics [33].

#### 4.1.3. PLS Analysis

Model construction was performed using multiple regression analysis based on the PLS method [60] within the context of 3D‐QSAR studies. The three‐dimensional descriptors derived from CoMSIA and CoMFA served as independent variables in the PLS regression, whereas the dependent variable was represented by the biological activity values, specifically pIC_50_. PLS analysis was conducted on the CoMSIA and CoMFA datasets. The model′s predictive performance was assessed through LOO cross‐validation. This method was employed for internal validation to identify the optimal number of components (*N*) that yielded the maximum cross‐validation coefficient(${{\text{r}}^{2}}_{\text{cv}}$) as delineated in Equation (1):

(1) q2=rcv2=1−∑n=1∞γp−γo2∑n=1∞γo−γo¯2

Detected and predicted activity values are represented by *γ*_*o*_ and *γ*_*p*_, moreover observed and predicted mean activity values in the training set are shown by $\bar{\gamma_{o}}$ and $\overset{\wedge }{\gamma_{p}}$ [61]. The predictive residual sum of squares (PRESS) is expressed by $\sum_{n=1}^{\infty }{\left(\gamma_{p}-\gamma_{o}\right)}^{2}$.

(2) r2=∑γo−γo¯γp−γp∧2∑γo−γo¯2×∑γp−γp∧2

The statistical confidence of the resulting models was assessed using the bootstrapping technique across 100 iterations [62–65]. To generate contour maps, CoMFA and CoMSIA methodologies were employed, utilizing the StDev∗Coeff field type along with the default contour levels.

#### 4.1.4. Verification of the QSAR Model

In the training set, robust internal validation yielded a high *q* ^2^ value; however, this was insufficient to substantiate the established models, necessitating external validation. In the validation study, the biological activities of molecules excluded from the training set were evaluated to determine the predictive capabilities of the 3D‐QSAR models (external validation). Based on the test set, the predictive correlation coefficient ${{\text{r}}^{2}}_{\text{pred}}$ (where ${{\text{r}}^{2}}_{\text{pred}}>0.6$) (12) was computed using the specified Equation (3):

(3) rpred2=SD−PRESSSD

The standard deviation (SD) is defined as the squared deviation between the mean biological activity of molecules in the test set and that of the training set. The PRESS is derived from the squared differences between the predicted and actual activities of the molecules [33].

#### 4.1.5. 3D‐QSAR Model Validation

Models exhibiting enhanced fitting capabilities generally possess lower SEE values, alongside greater *Q* ^2^, *R* ^2^, and *F* values. However, these statistical metrics alone are insufficient to comprehensively validate the efficacy of the proposed model. Therefore, supplementary techniques are necessary for further verification [66].

##### 4.1.5.1. External Validation Method

The following equation was employed in the external validation process of this study.

(4) Rext2=1−∑i=1ntestyi−y¯i2∑i=1ntestyi−y¯tr2

In this formula, the number of compounds in the test set is denoted as *n* *t* *e* *s* *t*, the average activity value of compounds in the training set is represented by ${\bar{y}}_{tr}$, and the experimental and predicted activity values of compounds in the test set are indicated by *y*_*i*_ and ${\bar{y}}_{i}$, respectively, in this formula. Generally, the model is considered stable and exhibits good predictive potential only when $R_{\mathrm{ext}}^{2}>0.5$ [57, 67].

##### 4.1.5.2. Confirmation With Independent Test Set

The predictive capabilities of the model were assessed using an independent test set that was not included in the model‐building process [37]. The stability and predictive power were evaluated through the quantification of the *q* ^2^ and *r* ^2^ metrics obtained from internal validation.

##### 4.1.5.3. *Y*‐randomization test

The *Y*‐randomization procedure is a robust method for validating a 3D‐QSAR model. By randomly altering the dependent variable, new 3D‐QSAR models are generated. The original model is considered valid if these new models exhibit low *q* ^2^ and *r* ^2^ values [68, 69].

### 4.2. Analyzing Docking Interactions in Specific Active Site

The MOE 2019 software (https://www.chemcomp.com/) was employed to explore the binding modes of screened derivatives within the active site of *H. pylori* urease. The crystal structure of *H. pylori* urease complexed with acetohydroxamic acid was obtained from the Protein Data Bank (PDB) [70–72] (PDB entry: 1E9Y). Protein selection criteria included minimal missing residues, no mutations, x‐ray crystallography, and cocrystal ligand acetohydroxamic acid (FDA‐approved), based on prior studies. The molecular structures of the compounds were constructed using the MOE Builder. After removing all bound water and ligands, polar hydrogen atoms were introduced to the protein [73, 74]. The enzyme underwent energy minimization using the Hamiltonian–Force Field–MMFF94x method in MOE [75] and was subsequently saved in PDB format. Docking was conducted using the triangle matcher approach, generating 70 poses [76]. The rigid protein method was applied to refine the GBWI/WSA DG after scoring across seven distinct poses. Finally, interactions between the compounds and enzyme complexes were analyzed using Discovery Studio Visualizer 4.0.

### 4.3. Design of New Analogues According to Ligand‐Based and Structure‐Based Studies

In research, the establishment of a comprehensive library of candidate compounds is crucial. The molecular structures derived from the pharmacophore, as depicted in the SAR presented in Figure 8, were sourced from the ZINC database (https://www.zinc.docking.org/) through pharmacophore‐based VS using MOE 2019. These compounds were initially provided in structured data file (SDF) format and subsequently converted to PDBQT format using PyRx 0.8 software, which is freely available at http://PyRx.sourceforge.net/downloads [75]. Following this conversion, blind docking‐based VS was performed on the candidate compounds.

### 4.4. Druglikeness Properties

Physicochemical properties, including molecular weight, flexibility, and hydrophobicity, significantly influence the behavior of molecules within a living organism. Achieving optimal bioavailability necessitates a suitable balance between solubility and permeability characteristics. To facilitate the development of effective oral drugs, Lipinski′s rule of five [77] was applied. The Molinspiration server (http://www.molinspiration.com/) was utilized to assess the molecular parameters of the compounds.

### 4.5. ADME Properties Prediction

The admetSAR online database (http://www.click2drug.org/directory-ADMET.html/admetSA) and the SwissADME server (http://www.swissadme.ch) were utilized to assess the ADME characteristics of the screened derivatives. The admetSAR platform provides web‐based query tools with an integrated molecular interface, enabling users to search the database using common names, IUPAC names, SMILES notation, or structural similarity [78]. The SwissADME server features a molecular sketcher powered by ChemAxon′s Marvin JS, which allows users to import, draw, and modify 2D chemical structures, subsequently converting them into a list of molecules. This interface supports modifications similar to regular text, permitting users to type or paste SMILES notation [79]. The screened structures were analyzed for interactions with metabolizing enzymes and their absorption characteristics.

### 4.6. In Silico Cardiotoxicity Prediction

The cardiotoxicity of the derivatives was assessed using the Pred‐hERG web server (http://www.labmol.com.br), a validated computational platform for predicting hERG channel inhibition. This method exhibits robust predictive performance, with reported sensitivity, accuracy, and specificity values nearing 90%. [75, 80, 81]

### 4.7. In Silico Cytotoxicity Activity Prediction

CLC‐Pred analysis was conducted using the online platform at http://www.way2drug.com/PASSonline to predict the cytotoxic effects of the derivatives based on their chemical structure. The results are presented as *P* *a* (probability of activity) and *P* *i* (probability of inactivity), both ranging from 0.000 to 1.000. It is important to note that *P* *a* and *P* *i* values are calculated independently and do not equal one. A *P* *a* value greater than 0.7 indicates a high likelihood that the compound will exhibit the predicted cytotoxic activity in experimental conditions. *P* *a* values between 0.5 and 0.7 suggest moderate probability, often reflecting structural dissimilarity to known drugs, and may imply reduced confidence in the predicted activity [82].

### 4.8. MD Simulations Study

MD simulations were performed under physiological conditions (37°C, 1 atm) in an explicit aqueous environment to investigate the interactions between the enzyme and selected ligand derivatives. The simulations employed the CHARMM27 force field and were conducted using NAMD (Git‐2018‐04‐24, Linux‐x86_64‐multicore‐CUDA; https://www.ks.uiuc.edu/), with input files generated using the MOE software [42, 83]. Three top‐scoring derivatives from the molecular docking analysis were selected for simulation. Prior to production runs, energy minimization of the enzyme–ligand complexes was carried out using the AMBER force field. Each complex was solvated in a rectangular water box (7 × 9 × 8 nm) sized to fully enclose the enzyme with sufficient space to ensure proper solvation and system stability. Partial atomic charges were assigned using MOE. The simulations were run for 100 ns, including an initial 1 ns of energy minimization. System trajectories were recorded every 20 ps and analyzed using the visual molecular dynamics (VMD) analyzer tools. All simulations were executed on AMD Ryzen 1950X 32‐core processors.

## Conflicts of Interest

The authors declare no conflicts of interest.

## Author Contributions

N. Shakour ran the experiments, analyzed the data, and wrote the original draft; A. Fayyaz ran the experiments and analyzed the data; F. Hadizadeh designed and ran the experiments and, analyzed the data; S. Sepehri designed and ran the characterization experiments, analyzed the data, and wrote the original draft as well as supervised the work, and all authors reviewed and edited the manuscript.

## Funding

This study was supported by the Ardabil University of Medical Sciences (10.13039/501100006662).

## Supporting information


**Supporting information** Additional supporting information can be found online in the Supporting Information section. Figures S1–S9 and Tables S1–S5: Supporting analyses, with authors responsible for their content. The authors clarify that the study did not utilize human‐derived data; as it is entirely in silico, the concepts of sex and specific tissue origin are not applicable. Additionally, no official cell lines were used, making an RRID unnecessary. This computational approach enables efficient drug candidate exploration, and the conclusions remain valid despite the absence of these details. Figure S1: Residual plots between predicted and investigational values for 3D‐QSAR models (train set [green] and test set [orange]). Figure S2 (a): Binding modes of compounds in urease active site (A) 1p, and (B) 1o. Figure S2 (b): Binding modes of compounds in urease active site (C) compound 2t, and (D) 1a. Figure S3: Binding mode of compound ZINC84668437 in urease enzyme. Figure S4: Binding mode of compound ZINC84669798 in urease enzyme. Figure S5: Binding mode of compound ZINC84669798 in urease enzyme. Figure S6: Binding mode of ZINC147991008 (a) and ZINC147991228 (b) in urease active site. Figure S7: The best conformation of ZINC000148002556 in urease enzyme. Figure S8: Potential energy plot of the ZINC244633273 (light blue), ZINC84669798 (purple), ZINC84668437 (green), 1p (red) as well as acetohydroxamic acid (dark blue) urease complexes during MD simulations. Figure S9: Numbers of hydrogen bonds formed between ZINC244633273, ZINC84669798, ZINC84668437, 1p, and acetohydroxamic acid with urease binding site residues during MD simulations. Table S1: Structures, predicted and investigational inhibitory activities lengthways with residuals of train set. Table S2: All screened ZINC ID candidates. Table S3: ADMET parameters of chosen derivatives predicted using SwissADME and admetSAR. Table S4: Cardiotoxicity estimate of selected compounds using PredhERG online server. Table S5: Brief overview of principal molecular interactions and docking scores in urease complexes generated using MOE.

## Data Availability

The authors confirmed that the data supporting the study′s conclusions are included in the article and its supporting information. Upon a reasonable request, the corresponding author will provide the raw data used to support the findings of this study.
