# Supplementary material for: Rational Design of Novel Inhibitors: Integrating 3D‐QSAR and Molecular Dynamics
Source: Biomed Res Int. 2026 Jan 31;2026:4138899. doi: 10.1155/bmri/4138899 (PMC12860141; doi:10.1155/bmri/4138899)
Supplement: Supplementary file 1 — Supporting information Additional supporting information can be found online in the Supporting Information section. Figures S1–S9 and Tables S1–S5: Supporting analyses, with authors responsible for their content. The authors clarify that the study did not utilize human‐derived data; as it is entirely in silico, the concepts of sex and specific tissue origin are not applicable. Additionally, no official cell lines were used, making an RRID unnecessary. This computational approach enables efficient drug candidate exploration, and the conclusions remain valid despite the absence of these details. Figure S1: Residual plots between predicted and investigational values for 3D‐QSAR models (train set [green] and test set [orange]). Figure S2 (a): Binding modes of compounds in urease active site (A) 1p, and (B) 1o. Figure S2 (b): Binding modes of compounds in urease active site (C) compound 2t, and (D) 1a. Figure S3: Binding mode of compound ZINC84668437 in urease enzyme. Figure S4: Binding mode of compound ZINC84669798 in urease enzyme. Figure S5: Binding mode of compound ZINC84669798 in urease enzyme. Figure S6: Binding mode of ZINC147991008 (a) and ZINC147991228 (b) in urease active site. Figure S7: The best conformation of ZINC000148002556 in urease enzyme. Figure S8: Potential energy plot of the ZINC244633273 (light blue), ZINC84669798 (purple), ZINC84668437 (green), 1p (red) as well as acetohydroxamic acid (dark blue) urease complexes during MD simulations. Figure S9: Numbers of hydrogen bonds formed between ZINC244633273, ZINC84669798, ZINC84668437, 1p, and acetohydroxamic acid with urease binding site residues during MD simulations. Table S1: Structures, predicted and investigational inhibitory activities lengthways with residuals of train set. Table S2: All screened ZINC ID candidates. Table S3: ADMET parameters of chosen derivatives predicted using SwissADME and admetSAR. Table S4: Cardiotoxicity estimate of selected compounds using PredhERG online server. Table S5 [file BMRI-2026-4138899-s001.zip › Table S2.docx]

| **Table S2.** All screened ZINC_ID candidates | | | | |
| --- | --- | --- | --- | --- |
| **No.** | ZINC_ID | **ZINC_ID** | **ZINC_ID** | **ZINC_ID** |
| **1** | ZINC000000124289 | ZINC000036153755 | ZINC000001018225 | ZINC000009361080 |
| **2** | ZINC000000148116 | ZINC000040267706 | ZINC000001018226 | ZINC000017180690 |
| **3** | ZINC000000151545 | ZINC000059168944 | ZINC000001018230 | ZINC000018141151 |
| **4** | ZINC000000151546 | ZINC000059168945 | ZINC000001018231 | ZINC000019808758 |
| **5** | ZINC000000208242 | ZINC000059168946 | ZINC000002182917 | ZINC000019850054 |
| **6** | ZINC000000208279 | ZINC000059168947 | ZINC000002183290 | ZINC000020131401 |
| **7** | ZINC000000208292 | ZINC000072416568 | ZINC000002183291 | ZINC000020588182 |
| **8** | ZINC000000208307 | ZINC000087587930 | ZINC000002183292 | ZINC000020932499 |
| **9** | ZINC000000208316 | ZINC000087588131 | ZINC000002183293 | ZINC000020932508 |
| **10** | ZINC000000208362 | ZINC000116478615 | ZINC000002183309 | ZINC000039932803 |
| **11** | ZINC000000208398 | ZINC000118648629 | ZINC000002862262 | ZINC000051376663 |
| **12** | ZINC000000337018 | ZINC000138490778 | ZINC000002874510 | ZINC000051376734 |
| **13** | ZINC000000390129 | ZINC000216756264 | ZINC000004667783 | ZINC000254791665 |
| **14** | ZINC000001398221 | ZINC000226299914 | ZINC000004772684 | ZINC000254860389 |
| **15** | ZINC000001398228 | ZINC000226299999 | ZINC000004772685 | ZINC000670428988 |
| **16** | ZINC000001401913 | ZINC000226300037 | ZINC000004857995 | ZINC000000383106 |
| **17** | ZINC000001586133 | ZINC000098087277 | ZINC000004858101 | ZINC000000383107 |
| **18** | ZINC000001586681 | ZINC000216756121 | ZINC000006719492 | ZINC000000383108 |
| **19** | ZINC000001587225 | ZINC000216756194 | ZINC000006757943 | ZINC000000384959 |
| **20** | ZINC000001617780 | ZINC000216756338 | ZINC000008392867 | ZINC000000384960 |
| **21** | ZINC000004753484 | ZINC000216756402 | ZINC000008392882 | ZINC000000385055 |
| **22** | ZINC000005533963 | ZINC000216756471 | ZINC000008392893 | ZINC000000385056 |
| **23** | ZINC000005721873 | ZINC000216756538 | ZINC000008392903 | ZINC000000385280 |
| **24** | ZINC000005952651 | ZINC000216756603 | ZINC000008392906 | ZINC000000385281 |
| **25** | ZINC000006645688 | ZINC000216756673 | ZINC000008392998 | ZINC000000385307 |
| **26** | ZINC000006669812 | ZINC000216756803 | ZINC000008715031 | ZINC000000385308 |
| **27** | ZINC000011585489 | ZINC000216756940 | ZINC000008715032 | ZINC000000385311 |
| **28** | ZINC000012341296 | ZINC000216757005 | ZINC000010311742 | ZINC000000385312 |
| **29** | ZINC000012341297 | ZINC000000058202 | ZINC000013727881 | ZINC000000385313 |
| **30** | ZINC000012341298 | ZINC000000383112 | ZINC000013732606 | ZINC000000385314 |
| **31** | ZINC000012417271 | ZINC000000383113 | ZINC000019909973 | ZINC000000385315 |
| **32** | ZINC000012579523 | ZINC000000383114 | ZINC000019909989 | ZINC000000385316 |
| **33** | ZINC000012579548 | ZINC000000383132 | ZINC000019909992 | ZINC000000385317 |
| **34** | ZINC000013118155 | ZINC000000384958 | ZINC000019909997 | ZINC000000385319 |
| **35** | ZINC000013150186 | ZINC000000385273 | ZINC000019910000 | ZINC000000385320 |
| **36** | ZINC000016264874 | ZINC000000385274 | ZINC000019910003 | ZINC000000612411 |
| **37** | ZINC000016265079 | ZINC000000385275 | ZINC000019910009 | ZINC000000613535 |
| **38** | ZINC000016265320 | ZINC000000385276 | ZINC000019910012 | ZINC000000632626 |
| **39** | ZINC000016267185 | ZINC000000385277 | ZINC000019910018 | ZINC000000632630 |
| **40** | ZINC000016732950 | ZINC000000385278 | ZINC000019910032 | ZINC000000632766 |
| **41** | ZINC000016732956 | ZINC000000385279 | ZINC000019910034 | ZINC000000632789 |
| **42** | ZINC000016733015 | ZINC000000385304 | ZINC000020723953 | ZINC000000641664 |
| **43** | ZINC000019624570 | ZINC000000612890 | ZINC000040284795 | ZINC000001018223 |
| **44** | ZINC000020529965 | ZINC000000613498 | ZINC000155587986 | ZINC000352348757 |
| **45** | ZINC000022478484 | ZINC000001018224 | ZINC000255187249 | ZINC000358885845 |
| **46** | ZINC000026966105 | ZINC000002861303 | ZINC000000344815 | ZINC000427281577 |
| **47** | ZINC000027642555 | ZINC000002862008 | ZINC000000344816 | ZINC000435799064 |
| **48** | ZINC000031891201 | ZINC000004114394 | ZINC000000457735 | ZINC000462656315 |
| **49** | ZINC000031891204 | ZINC000005032326 | ZINC000000520328 | ZINC000472207590 |
| **50** | ZINC000031891219 | ZINC000005032327 | ZINC000000988403 | ZINC000485486024 |
| **51** | ZINC000031891229 | ZINC000005934306 | ZINC000001240468 | ZINC000485487299 |
| **52** | ZINC000031891249 | ZINC000008715038 | ZINC000001240469 | ZINC000486231777 |
| **53** | ZINC000031891253 | ZINC000009547038 | ZINC000001241083 | ZINC000509034896 |
| **54** | ZINC000031891256 | ZINC000012377876 | ZINC000001241184 | ZINC000509039682 |
| **55** | ZINC000032250573 | ZINC000013532602 | ZINC000001241185 | ZINC000509041392 |
| **56** | ZINC000033162025 | ZINC000013692122 | ZINC000001241284 | ZINC000509068190 |
| **57** | ZINC000042379509 | ZINC000019909976 | ZINC000001243715 | ZINC000515208468 |
| **58** | ZINC000082533511 | ZINC000026670105 | ZINC000001243716 | ZINC000522000135 |
| **59** | ZINC000218439335 | ZINC000034597316 | ZINC000001246216 | ZINC000538708154 |
| **60** | ZINC000218449120 | ZINC000036153702 | ZINC000001247712 | ZINC000592349338 |
| **61** | ZINC000224971930 | ZINC000092249775 | ZINC000001248984 | ZINC000634548430 |
| **62** | ZINC000224972174 | ZINC000095457508 | ZINC000001252211 | ZINC000634548482 |
| **63** | ZINC000252679289 | ZINC000095501527 | ZINC000001254347 | ZINC000634548620 |
| **64** | ZINC000252679338 | ZINC000095507488 | ZINC000001257952 | ZINC000634548640 |
| **65** | ZINC000254293602 | ZINC000095508477 | ZINC000001261480 | ZINC000634548773 |
| **66** | ZINC000254485232 | ZINC000095508478 | ZINC000001261649 | ZINC000634548802 |
| **67** | ZINC000254498858 | ZINC000096008311 | ZINC000001272310 | ZINC000634548936 |
| **68** | ZINC000254531943 | ZINC000097047705 | ZINC000001275583 | ZINC000634552958 |
| **69** | ZINC000254566155 | ZINC000097083521 | ZINC000001279658 | ZINC000634553307 |
| **70** | ZINC000254566240 | ZINC000097088563 | ZINC000001293721 | ZINC000671617821 |
| **71** | ZINC000254567367 | ZINC000097114219 | ZINC000001299371 | ZINC000683737762 |
| **72** | ZINC000254589453 | ZINC000097114229 | ZINC000001300528 | ZINC000683924842 |
| **73** | ZINC000254622989 | ZINC000170593971 | ZINC000001301128 | ZINC000684093359 |
| **74** | ZINC000254700856 | ZINC000170612185 | ZINC000001301850 | ZINC000684886713 |
| **75** | ZINC000254706403 | ZINC000171542693 | ZINC000001302405 | ZINC000685035250 |
| **76** | ZINC000254762796 | ZINC000172916883 | ZINC000001305413 | ZINC000822288503 |
| **77** | ZINC000254832912 | ZINC000180015669 | ZINC000001305604 | ZINC000871746414 |
| **78** | ZINC000256027773 | ZINC000195434412 | ZINC000001307146 | ZINC000871747159 |
| **79** | ZINC000256027890 | ZINC000195437383 | ZINC000001307175 | ZINC001117128888 |
| **80** | ZINC000256027941 | ZINC000244633273 | ZINC000001455422 | ZINC001121269865 |
| **81** | ZINC000256027972 | ZINC000244884274 | ZINC000001455423 | ZINC001121270436 |
| **82** | ZINC000256028051 | ZINC000264215865 | ZINC000001473695 | ZINC001121769351 |
| **83** | ZINC000256028052 | ZINC000264234949 | ZINC000001475943 | ZINC001121769839 |
| **84** | ZINC000256055363 | ZINC000278600119 | ZINC000001835411 | ZINC001165354112 |
| **85** | ZINC000256055450 | ZINC000279089484 | ZINC000003886218 | ZINC001262853346 |
| **86** | ZINC000256055467 | ZINC000281531181 | ZINC000004205503 | ZINC001263011985 |
| **87** | ZINC000256055550 | ZINC000282297463 | ZINC000004899331 | ZINC001321277814 |
| **88** | ZINC000256055653 | ZINC000082409812 | ZINC000005731529 | ZINC001327163922 |
| **89** | ZINC000256055745 | ZINC000082409814 | ZINC000005810228 | ZINC001337936985 |
| **90** | ZINC000256055795 | ZINC000084378739 | ZINC000005900458 | ZINC001337937830 |
| **91** | ZINC000256055878 | ZINC000084378741 | ZINC000005911409 | ZINC001338076477 |
| **92** | ZINC000263629291 | ZINC000085271859 | ZINC000005911686 | ZINC001338077192 |
| **93** | ZINC000584905795 | ZINC000086194610 | ZINC000005968891 | ZINC001338077194 |
| **94** | ZINC000584905796 | ZINC000095097451 | ZINC000005968895 | ZINC001339439600 |
| **95** | ZINC000618254478 | ZINC000095946096 | ZINC000005969754 | ZINC001339555418 |
| **96** | ZINC000618254493 | ZINC000096032001 | ZINC000005981742 | ZINC001350441721 |
| **97** | ZINC000618254664 | ZINC000096032172 | ZINC000005981881 | ZINC001350443921 |
| **98** | ZINC000618254904 | ZINC000096032990 | ZINC000005982053 | ZINC001355334580 |
| **99** | ZINC000618254950 | ZINC000096321491 | ZINC000005982054 | ZINC001355334625 |
| **100** | ZINC000618254980 | ZINC000096516287 | ZINC000005998841 | ZINC001355335188 |
| **101** | ZINC000000383127 | ZINC000096516288 | ZINC000006056315 | ZINC001652797828 |
| **102** | ZINC000000385119 | ZINC000096517292 | ZINC000006199719 | ZINC001772710533 |
| **103** | ZINC000001017783 | ZINC000096527936 | ZINC000006499588 | ZINC001772710536 |
| **104** | ZINC000001657453 | ZINC000096527964 | ZINC000006556077 | ZINC000004072961 |
| **105** | ZINC000001657454 | ZINC000096527966 | ZINC000006556595 | ZINC000004072962 |
| **106** | ZINC000001657455 | ZINC000098095314 | ZINC000006557425 | ZINC000004072964 |
| **107** | ZINC000001657458 | ZINC000100211884 | ZINC000006557718 | ZINC000004088820 |
| **108** | ZINC000001704190 | ZINC000101530864 | ZINC000006557721 | ZINC000004088831 |
| **109** | ZINC000001704193 | ZINC000104558531 | ZINC000007601452 | ZINC000004088894 |
| **110** | ZINC000001704196 | ZINC000104643595 | ZINC000007601463 | ZINC000004088901 |
| **111** | ZINC000001719294 | ZINC000104644977 | ZINC000007601464 | ZINC000004088908 |
| **112** | ZINC000002024436 | ZINC000127972776 | ZINC000007601485 | ZINC000004088911 |
| **113** | ZINC000002216086 | ZINC000143169947 | ZINC000007601538 | ZINC000008685917 |
| **114** | ZINC000002219176 | ZINC000143170167 | ZINC000007601539 | ZINC000008781572 |
| **115** | ZINC000002338986 | ZINC000169726330 | ZINC000007601545 | ZINC000012955643 |
| **116** | ZINC000002346071 | ZINC000169726384 | ZINC000007601815 | ZINC000016363305 |
| **117** | ZINC000002346073 | ZINC000005369061 | ZINC000007601993 | ZINC000020583115 |
| **118** | ZINC000003848413 | ZINC000005369064 | ZINC000007602000 | ZINC000020588798 |
| **119** | ZINC000004487466 | ZINC000005369070 | ZINC000007602087 | ZINC000080844478 |
| **120** | ZINC000004487480 | ZINC000005375114 | ZINC000008338009 | ZINC000109609768 |
| **121** | ZINC000004487485 | ZINC000005375131 | ZINC000008474777 | ZINC000109609770 |
| **122** | ZINC000004487532 | ZINC000005375158 | ZINC000008494689 | ZINC000147991008 |
| **123** | ZINC000004487534 | ZINC000005375180 | ZINC000008496031 | ZINC000147991228 |
| **124** | ZINC000004487537 | ZINC000005375203 | ZINC000008496651 | ZINC000148002556 |
| **125** | ZINC000004487538 | ZINC000005375222 | ZINC000008496667 | ZINC000148002776 |
| **126** | ZINC000004487541 | ZINC000005416339 | ZINC000008496669 | ZINC000153358549 |
| **127** | ZINC000005533617 | ZINC000005416355 | ZINC000008496672 | ZINC000153358659 |
| **128** | ZINC000005541348 | ZINC000005416359 | ZINC000008550100 | ZINC000153359128 |
| **129** | ZINC000006661560 | ZINC000005416706 | ZINC000008551030 | ZINC000153359373 |
| **130** | ZINC000006668089 | ZINC000005416709 | ZINC000640955950 | ZINC001192936133 |
| **131** | ZINC000006668613 | ZINC000005416726 | ZINC000640989429 | ZINC001192936134 |
| **132** | ZINC000006668614 | ZINC000015774086 | ZINC000640990424 | ZINC001192936436 |
| **133** | ZINC000006668735 | ZINC000015774092 | ZINC000641004877 | ZINC001192936452 |
| **134** | ZINC000006668736 | ZINC000015774094 | ZINC000641013842 | ZINC001192936457 |
| **135** | ZINC000006668739 | ZINC000017780325 | ZINC000641013850 | ZINC001192936483 |
| **136** | ZINC000006818043 | ZINC000017780800 | ZINC000641039329 | ZINC001192936507 |
| **137** | ZINC000006818166 | ZINC000017822708 | ZINC000641047347 | ZINC001192936510 |
| **138** | ZINC000006818190 | ZINC000017920363 | ZINC000641047693 | ZINC001192936511 |
| **139** | ZINC000007737841 | ZINC000018030827 | ZINC000641047874 | ZINC001192936525 |
| **140** | ZINC000008617477 | ZINC000018166877 | ZINC000641056133 | ZINC001192936530 |
| **141** | ZINC000009286887 | ZINC000026982083 | ZINC000641061832 | ZINC001192936531 |
| **142** | ZINC000009286889 | ZINC000033897913 | ZINC000641066419 | ZINC001192936540 |
| **143** | ZINC000009286896 | ZINC000033897914 | ZINC000641072099 | ZINC001192936771 |
| **144** | ZINC000009341537 | ZINC001078341091 | ZINC000641074735 | ZINC001192936781 |
| **145** | ZINC000009421321 | ZINC001078341092 | ZINC000641074736 | ZINC001192936824 |
| **146** | ZINC000012388934 | ZINC001435795187 | ZINC000641077378 | ZINC001192936827 |
| **147** | ZINC000012409907 | ZINC000771966777 | ZINC000641080596 | ZINC001192936846 |
| **148** | ZINC000013723843 | ZINC000754363411 | ZINC000641080602 | ZINC001192936874 |
| **149** | ZINC000013727287 | ZINC000640945575 | ZINC000641095173 | ZINC001192936901 |
| **150** | ZINC000015080775 | ZINC000575473227 | ZINC000641095416 | ZINC001192936910 |
| **151** | ZINC000017353482 | ZINC000203462565 | ZINC000641096327 | ZINC001192937037 |
| **152** | ZINC000019737419 | ZINC000103221855 | ZINC000641097369 | ZINC001192937042 |
| **153** | ZINC000019772742 | ZINC000051629097 | ZINC000641097411 | ZINC001192937046 |
| **154** | ZINC000020159025 | ZINC000051629095 | ZINC000641097833 | ZINC001192937073 |
| **155** | ZINC000020159213 | ZINC000051629094 | ZINC000641112983 | ZINC001192937075 |
| **156** | ZINC000020159215 | ZINC000051629092 | ZINC000641124426 | ZINC001192937080 |
| **157** | ZINC000020159217 | ZINC000051629090 | ZINC000641132734 | ZINC001192937082 |
| **158** | ZINC000020272933 | ZINC000051629089 | ZINC000641133310 | ZINC001192937126 |
| **159** | ZINC000020272935 | ZINC000013691161 | ZINC000641158985 | ZINC001192937193 |
| **160** | ZINC000020272939 | ZINC000005748595 | ZINC000641172837 | ZINC001192937194 |
| **161** | ZINC000020606033 | ZINC000000505445 | ZINC000656437043 | ZINC001192937210 |
| **162** | ZINC000020727869 | ZINC000051629080 | ZINC000656437151 | ZINC001192937229 |
| **163** | ZINC000021526223 | ZINC000109612123 | ZINC000656437185 | ZINC001192937257 |
| **164** | ZINC000021526235 | ZINC000051629078 | ZINC000656437471 | ZINC001192937299 |
| **165** | ZINC000022504005 | ZINC000109612126 | ZINC000656437497 | ZINC001192937304 |
| **166** | ZINC000022504345 | ZINC001772571850 | ZINC000656437831 | ZINC001192937316 |
| **167** | ZINC000026548202 | ZINC001772605453 | ZINC000656459546 | ZINC001192937379 |
| **168** | ZINC000031807277 | ZINC000005032360 | ZINC000656460032 | ZINC001192938355 |
| **169** | ZINC000032012184 | ZINC000000531046 | ZINC000656460033 | ZINC001192938390 |
| **170** | ZINC000032012672 | ZINC000002356517 | ZINC000656460200 | ZINC001192938395 |
| **171** | ZINC000032014502 | ZINC000036723685 | ZINC000656460559 | ZINC001192938480 |
| **172** | ZINC000033846226 | ZINC000069910550 | ZINC000656460703 | ZINC001219697257 |
| **173** | ZINC000036153654 | ZINC000069910551 | ZINC000656460704 | ZINC001219714742 |
| **174** | ZINC000036353231 | ZINC000007601998 | ZINC000656460727 | ZINC001219720676 |
| **175** | ZINC000036358512 | ZINC000007602019 | ZINC000656460728 | ZINC001220059080 |
| **176** | ZINC000036359859 | ZINC000007602026 | ZINC000656460941 | ZINC001220874209 |
| **177** | ZINC000044893247 | ZINC000007602031 | ZINC000656461086 | ZINC001221346553 |
| **178** | ZINC000044900646 | ZINC000007602060 | ZINC000656461097 | ZINC001221379870 |
| **179** | ZINC000057332534 | ZINC000007602062 | ZINC000656461098 | ZINC001221379871 |
| **180** | ZINC000072404911 | ZINC000007602065 | ZINC000656461099 | ZINC001254032147 |
| **181** | ZINC000073636211 | ZINC000007602068 | ZINC000656461103 | ZINC001254126773 |
| **182** | ZINC000079480226 | ZINC000007602072 | ZINC000656464903 | ZINC001255145075 |
| **183** | ZINC000079487933 | ZINC000007602076 | ZINC000656464904 | ZINC001570001177 |
| **184** | ZINC000079495384 | ZINC000007602090 | ZINC000656465241 | ZINC001570001726 |
| **185** | ZINC000095475709 | ZINC000007602114 | ZINC000656465242 | ZINC000084668434 |
| **186** | ZINC000097466819 | ZINC000007602119 | ZINC000881271121 | ZINC000084668435 |
| **187** | ZINC000216755849 | ZINC000007602132 | ZINC000881316928 | ZINC000084668436 |
| **188** | ZINC000216760761 | ZINC000007602145 | ZINC000881379121 | ZINC000084668437 |
| **189** | ZINC000216931293 | ZINC000007602159 | ZINC000900609897 | ZINC000084668840 |
| **190** | ZINC000216931355 | ZINC000007602184 | ZINC000001035377 | ZINC000084669111 |
| **191** | ZINC000226299814 | ZINC000007602209 | ZINC000001257750 | ZINC000084669457 |
| **192** | ZINC000226299825 | ZINC000007602233 | ZINC000001299977 | ZINC000084669458 |
| **193** | ZINC000226299833 | ZINC000007602376 | ZINC000005611806 | ZINC000084669795 |
| **194** | ZINC000226773679 | ZINC000008253402 | ZINC000005828988 | ZINC000084669796 |
| **195** | ZINC000226773709 | ZINC000008253445 | ZINC000005998118 | ZINC000084669797 |
| **196** | ZINC000226774148 | ZINC000008253482 | ZINC000006228023 | ZINC000084669798 |
| **197** | ZINC000226774199 | ZINC000008253699 | ZINC000007365451 | ZINC000084669816 |
| **198** | ZINC000226775097 | ZINC000008254373 | ZINC000007601769 | ZINC000084670159 |
| **199** | ZINC000252624917 | ZINC000008324125 | ZINC000007601775 | ZINC000084703949 |
| **200** | ZINC000584614463 | ZINC000008324605 | ZINC000007601777 | ZINC000084740522 |
| **201** | ZINC000000156765 | ZINC000008324606 | ZINC000007601781 | ZINC000084759220 |
| **202** | ZINC000000382755 | ZINC000008324759 | ZINC000007601787 | ZINC000603248515 |
| **203** | ZINC000001101801 | ZINC000008324960 | ZINC000007601790 | ZINC000640833660 |
| **204** | ZINC000001101802 | ZINC000008325158 | ZINC000007601793 | ZINC000640833750 |
| **205** | ZINC000001323929 | ZINC000008325324 | ZINC000007601796 | ZINC000640842367 |
| **206** | ZINC000001332993 | ZINC000008325617 | ZINC000007601798 | ZINC000640859761 |
| **207** | ZINC000001430876 | ZINC000008338016 | ZINC000007601805 | ZINC000640891241 |
| **208** | ZINC000001576386 | ZINC000008378285 | ZINC000007601808 | ZINC000640935349 |
| **209** | ZINC000002521310 | ZINC000008418805 | ZINC000007601811 | ZINC000640942538 |
| **210** | ZINC000002569113 | ZINC000008469578 | ZINC000007601817 | ZINC000640946695 |
| **211** | ZINC000002638976 | ZINC000008469651 | ZINC000007601845 | ZINC000640948049 |
| **212** | ZINC000002684328 | ZINC000008469890 | ZINC000007601856 | ZINC000640948050 |
| **213** | ZINC000002788808 | ZINC000008469963 | ZINC000007601863 | ZINC000640948413 |
| **214** | ZINC000004263035 | ZINC000008470169 | ZINC000007601981 | ZINC000640948416 |
| **215** | ZINC000004294300 | ZINC000008471027 | ZINC000007601984 | ZINC000640955922 |
| **216** | ZINC000004294302 | ZINC000008473125 | ZINC000001481815 | ZINC000074934359 |
| **217** | ZINC000004339111 | ZINC000008473564 | ZINC000504466265 | ZINC000087587890 |
| **218** | ZINC000004453498 | ZINC000008474781 | ZINC001157666063 | ZINC000003723237 |
| **219** | ZINC000004704615 | ZINC000008493041 | ZINC001160645100 | ZINC000063846043 |
| **220** | ZINC000004761200 | ZINC000008493045 | ZINC001160654779 | ZINC000065380883 |
| **221** | ZINC000004761202 | ZINC000008493744 | ZINC001161038749 | ZINC000065381721 |
| **222** | ZINC000004966588 | ZINC000008494370 | ZINC001161115138 | ZINC000065397070 |
| **223** | ZINC000004966597 | ZINC000008494697 | ZINC001161138665 | ZINC000065451281 |
| **224** | ZINC000005000490 | ZINC000008495214 | ZINC001161138666 | ZINC000065508358 |
| **225** | ZINC000005578428 | ZINC000008496037 | ZINC001161418717 | ZINC000065508554 |
| **226** | ZINC000005670085 | ZINC000008496880 | ZINC001161493907 | ZINC000065526350 |
| **227** | ZINC000005730872 | ZINC000008534143 | ZINC001161508098 | ZINC000065533743 |
| **228** | ZINC000005749026 | ZINC000008535231 | ZINC001161508099 | ZINC000067935399 |
| **229** | ZINC000005749031 | ZINC000008548500 | ZINC001192928711 | ZINC000069570733 |
| **230** | ZINC000005880920 | ZINC000008550102 | ZINC001192928722 | ZINC000078923104 |
| **231** | ZINC000005881295 | ZINC000008551032 | ZINC001192928758 | ZINC000078950872 |
| **232** | ZINC000006175328 | ZINC000016778095 | ZINC001192928794 | ZINC000096868775 |
| **233** | ZINC000006176116 | ZINC000016778119 | ZINC001192928795 | ZINC000104157833 |
| **234** | ZINC000006185651 | ZINC000016778121 | ZINC001192929464 | ZINC000252494581 |
| **235** | ZINC000006219514 | ZINC000016778123 | ZINC001192929548 | ZINC000306251927 |
| **236** | ZINC000006320700 | ZINC000016778125 | ZINC001192929899 | ZINC000306374308 |
| **237** | ZINC000006320702 | ZINC000016778127 | ZINC001192929913 | ZINC000306817955 |
| **238** | ZINC000006480099 | ZINC000016778129 | ZINC001192929976 | ZINC000307262290 |
| **239** | ZINC000006605311 | ZINC000016778131 | ZINC001192929978 | ZINC000307262291 |
| **240** | ZINC000006668365 | ZINC000016778133 | ZINC001192930199 | ZINC000000301667 |
| **241** | ZINC000006816975 | ZINC000022111036 | ZINC001192930209 | ZINC000000967863 |
| **242** | ZINC000006828374 | ZINC000022111151 | ZINC001192930222 | ZINC000062316438 |
| **243** | ZINC000008211978 | ZINC000252622059 | ZINC001192930233 | ZINC000072155067 |
| **244** | ZINC000008212004 | ZINC000252627672 | ZINC001192930272 | ZINC000072155086 |
| **245** | ZINC000008244856 | ZINC000252634855 | ZINC001192930533 | ZINC000072186885 |
| **246** | ZINC000008600485 | ZINC000254352774 | ZINC001192930600 | ZINC000072221068 |
| **247** | ZINC000008600487 | ZINC000261148381 | ZINC001192930942 | ZINC000078210675 |
| **248** | ZINC000008704654 | ZINC000000987391 | ZINC001192930959 | ZINC000078224415 |
| **249** | ZINC000008913863 | ZINC000001071228 | ZINC001192935997 | ZINC000082258750 |
| **250** | ZINC000009738567 | ZINC000001417947 | ZINC001192936000 | ZINC000082406755 |
| **251** | ZINC000011919854 | ZINC000001417949 | ZINC001192936001 | ZINC000082406756 |
| **252** | ZINC000011919855 | ZINC000001470795 | ZINC001192936017 | ZINC000068576213 |
| **253** | ZINC000013142733 | ZINC000001576565 | ZINC001192936018 | ZINC000075844713 |
| **254** | ZINC000013564625 | ZINC000001576571 | ZINC001192936019 | ZINC000082160294 |
| **255** | ZINC000016890641 | ZINC000001647435 | ZINC001192936107 | ZINC000091305590 |
| **256** | ZINC000016951351 | ZINC000001653220 | ZINC001192936108 | ZINC000095908509 |
| **257** | ZINC000016957707 | ZINC000001666567 | ZINC001192936115 | ZINC000096530451 |
| **258** | ZINC000016957739 | ZINC000003165123 | ZINC001192936130 | ZINC000096530452 |
| **259** | ZINC000016957843 | ZINC000003900784 | ZINC000057218682 | ZINC000096530455 |
| **260** | ZINC000018043370 | ZINC000004342740 | ZINC000036559091 | ZINC000051783987 |
| **261** | ZINC000019087795 | ZINC000004430629 | ZINC000039268776 | ZINC000054931761 |
| **262** | ZINC000026466997 | ZINC000004552268 | ZINC000039268778 | ZINC000057218662 |
| **263** | ZINC000026479705 | ZINC000005344138 | ZINC000039274343 | ZINC000057218686 |
| **264** | ZINC000030714509 | ZINC000005380621 | ZINC000039632300 | ZINC000057218709 |
| **265** | ZINC000032006663 | ZINC000005462261 | ZINC000048446435 | ZINC000057218714 |
| **266** | ZINC000033749160 | ZINC000005544260 | ZINC000050213513 | ZINC000057218729 |
| **267** | ZINC000035109328 | ZINC000005778133 | ZINC000050213554 | ZINC000057218730 |
| **268** | ZINC000036047248 | ZINC000006378775 | ZINC000050215244 | ZINC000058037999 |
| **269** | ZINC000039331477 | ZINC000006494791 | ZINC000052221654 | ZINC000062152630 |
| **270** | ZINC000043497158 | ZINC000006499692 | ZINC000022688833 | ZINC000035185402 |
| **271** | ZINC000043497161 | ZINC000006862763 | ZINC000023289000 | ZINC000036379848 |
| **272** | ZINC000043501251 | ZINC000012428229 | ZINC000024565392 | ZINC000051783986 |
| **273** | ZINC000048229497 | ZINC000017322468 | ZINC000026422982 | ZINC000018123155 |
| **274** | ZINC000033436116 |  | | |
