# Supplementary material for: Rational Design of Novel Inhibitors: Integrating 3D‐QSAR and Molecular Dynamics
Source: Biomed Res Int. 2026 Jan 31;2026:4138899. doi: 10.1155/bmri/4138899 (PMC12860141; doi:10.1155/bmri/4138899)
Supplement: Supplementary file 1 — Supporting information Additional supporting information can be found online in the Supporting Information section. Figures S1–S9 and Tables S1–S5: Supporting analyses, with authors responsible for their content. The authors clarify that the study did not utilize human‐derived data; as it is entirely in silico, the concepts of sex and specific tissue origin are not applicable. Additionally, no official cell lines were used, making an RRID unnecessary. This computational approach enables efficient drug candidate exploration, and the conclusions remain valid despite the absence of these details. Figure S1: Residual plots between predicted and investigational values for 3D‐QSAR models (train set [green] and test set [orange]). Figure S2 (a): Binding modes of compounds in urease active site (A) 1p, and (B) 1o. Figure S2 (b): Binding modes of compounds in urease active site (C) compound 2t, and (D) 1a. Figure S3: Binding mode of compound ZINC84668437 in urease enzyme. Figure S4: Binding mode of compound ZINC84669798 in urease enzyme. Figure S5: Binding mode of compound ZINC84669798 in urease enzyme. Figure S6: Binding mode of ZINC147991008 (a) and ZINC147991228 (b) in urease active site. Figure S7: The best conformation of ZINC000148002556 in urease enzyme. Figure S8: Potential energy plot of the ZINC244633273 (light blue), ZINC84669798 (purple), ZINC84668437 (green), 1p (red) as well as acetohydroxamic acid (dark blue) urease complexes during MD simulations. Figure S9: Numbers of hydrogen bonds formed between ZINC244633273, ZINC84669798, ZINC84668437, 1p, and acetohydroxamic acid with urease binding site residues during MD simulations. Table S1: Structures, predicted and investigational inhibitory activities lengthways with residuals of train set. Table S2: All screened ZINC ID candidates. Table S3: ADMET parameters of chosen derivatives predicted using SwissADME and admetSAR. Table S4: Cardiotoxicity estimate of selected compounds using PredhERG online server. Table S5 [file BMRI-2026-4138899-s001.zip › Table S3.docx]

| **No.** | **GI** | **BBB** | **P-gpS** | **LogS** | **Cyp1A2** | **Cyp2C19** | **Cyp2C9** | **Cyp2D6** | **Cyp3A4** | **Carc.** |
| --- | --- | --- | --- | --- | --- | --- | --- | --- | --- | --- |
| **ZINC84668437** | H. | No | No | Sol. | No | No | No | No | No | No |
| **ZINC84669798** | H. | No | No | Sol. | No | No | No | No | No | No |
| **ZINC244633273** | H. | No | No | Sol. | No | No | No | No | No | No |
| **ZINC147991008** | H. | No | No | Sol. | No | No | No | No | No | No |
| **ZINC147991228** | H. | No | No | Sol. | No | No | No | No | No | No |
| **ZINC000148002556** | H. | No | No | Sol. | No | No | No | No | No | No |

P-gpS: P-gp Substrate; Carc.: Carcinogenicity; H.: High; Sol.: Solubility.
